# Dimethylarginine dimethylaminohydrolase 1 protects PM_2.5_ exposure-induced lung injury in mice by repressing inflammation and oxidative stress

**DOI:** 10.1186/s12989-022-00505-7

**Published:** 2022-10-14

**Authors:** Junling Gao, Tong Lei, Hongyun Wang, Kai Luo, Yuanli Wang, Bingqing Cui, Zhuoran Yu, Xiaoqi Hu, Fang Zhang, Yingjie Chen, Wenjun Ding, Zhongbing Lu

**Affiliations:** 1 College of Life Sciences, University of Chinese Academy of Sciences, 19A Yuquanlu, Beijing, 100049 China; 2 Cardiac Regeneration and Ageing Lab, Institute of Cardiovascular Sciences, School of Life Sciences, Shanghai University, Shanghai, 200444 China; 3 Department of Physiology and Biophysics, University of Mississippi Medical Center, Jackson, MS 39216 USA

**Keywords:** DDAH1, PM_2.5_, Lung injury, Inflammation, Oxidative stress

## Abstract

**Background:**

Airborne fine particulate matter with aerodynamic diameter ≤ 2.5 μm (PM_2.5_) pollution is associated with the prevalence of respiratory diseases, including asthma, bronchitis and chronic obstructive pulmonary disease. In patients with those diseases, circulating asymmetric dimethylarginine (ADMA) levels are increased, which contributes to airway nitric oxide deficiency, oxidative stress and inflammation. Overexpression of dimethylarginine dimethylaminohydrolase 1 (DDAH1), an enzyme degrading ADMA, exerts protective effects in animal models. However, the impact of DDAH1/ADMA on PM_2.5_-induced lung injury has not been investigated.

**Methods:**

*Ddah1*^−/−^ and DDAH1-transgenic mice, as well as their respective wild-type (WT) littermates, were exposed to either filtered air or airborne PM_2.5_ (mean daily concentration ~ 50 µg/m^3^) for 6 months through a whole-body exposure system. Mice were also acutely exposed to 10 mg/kg PM_2.5_ and/or exogenous ADMA (2 mg/kg) via intratracheal instillation every other day for 2 weeks. Inflammatory response, oxidative stress and related gene expressions in the lungs were examined. In addition, RAW264.7 cells were exposed to PM_2.5_ and/or ADMA and the changes in intracellular oxidative stress and inflammatory response were determined.

**Results:**

*Ddah1*^−/−^ mice developed more severe lung injury than WT mice after long-term PM_2.5_ exposure, which was associated with greater induction of pulmonary oxidative stress and inflammation. In the lungs of PM_2.5_-exposed mice, *Ddah1* deficiency increased protein expression of p-p65, iNOS and Bax, and decreased protein expression of Bcl-2, SOD1 and peroxiredoxin 4. Conversely, DDAH1 overexpression significantly alleviated lung injury, attenuated pulmonary oxidative stress and inflammation, and exerted opposite effects on those proteins in PM_2.5_-exposed mice. In addition, exogenous ADMA administration could mimic the effect of *Ddah1* deficiency on PM_2.5_-induced lung injury, oxidative stress and inflammation. In PM_2.5_-exposed macrophages, ADMA aggravated the inflammatory response and oxidative stress in an iNOS-dependent manner.

**Conclusion:**

Our data revealed that DDAH1 has a marked protective effect on long-term PM_2.5_ exposure-induced lung injury.

**Supplementary Information:**

The online version contains supplementary material available at 10.1186/s12989-022-00505-7.

## Background

Ambient air pollution, specifically environmental fine particulate matter (PM_2.5_, aerodynamic diameter ≤ 2.5 µm), is a major threat to public health [1, 2]. There is an unequivocal association between PM_2.5_ pollution and respiratory diseases [3]. Epidemiological studies have shown that long-term exposure to high concentrations of PM_2.5_ increases the risk of respiratory diseases, including asthma [4], bronchitis [5] and chronic obstructive pulmonary disease (COPD) [6]. Due to its size, after inhalation, PM_2.5_ can pass through the nose hair filtration stage, enter the lower respiratory tract and even penetrate the alveolar space [7]. PM_2.5_ deposited in lung alveoli causes lung injury by promoting reactive oxygen species (ROS) production and proinflammatory cytokine release [8]. Animal experiments have demonstrated that PM_2.5_ exposure increases pulmonary oxidative stress and inflammation in a dose-dependent manner [9, 10]. Although transcriptomic and proteomic analyses have been performed to investigate the precise underlying mechanism of PM_2.5_-induced lung injury [10, 11], it is still necessary to identify potential therapeutic targets for attenuating PM_2.5_-associated pulmonary diseases.

Asymmetrical dimethylarginine (ADMA) is a natural analog of l-arginine (l-Arg) and inhibits nitric oxide synthase (NOS) activity by competing with l-Arg. Emerging evidence indicates that ADMA plays a critical role in the pathogenesis of respiratory diseases through regulating nitric oxide (NO) production in vivo [12]. In mammal, ADMA is mainly degraded by dimethylarginine dimethylaminohydrolase (DDAH), which has two isoforms: DDAH1 and DDAH2. Increased lung ADMA levels and decreased DDAH expression were observed in allergen challenge-induced airway inflammation animal models [13], and overexpression of DDAH1 could attenuate airway inflammation induced by ovalbumin, agricultural organic dust extract, or house dust mites [14–16]. Our previous study also demonstrated that both DDAH1 knockdown and overexpression could attenuate PM_2.5_-induced cell death in A549 cells [17]. However, the in vivo effect of ADMA/DDAH1 on PM_2.5_ exposure-induced lung injury has not been recognized. To address this question, *Ddah1*-deficient *(Ddah1*^−/−^) and human DDAH1 transgenic mice (DDAH1-TG), as well as their respective wild-type (WT) littermates were exposed to either ambient PM_2.5_ or filtered air (FA) for 3–6 months through a whole-body PM exposure system. Mice were also acutely exposed to PM_2.5_ and/or ADMA via intratracheal instillation to determine the in vivo effect of ADMA on lung injury. Since exogenous ADMA had no effect on PM_2.5_-exposed A549 cells [17], the in vitro synergistic effect of PM_2.5_ and ADMA were investigated in RAW264.7 cells.

## Results

**Fig. 1:**
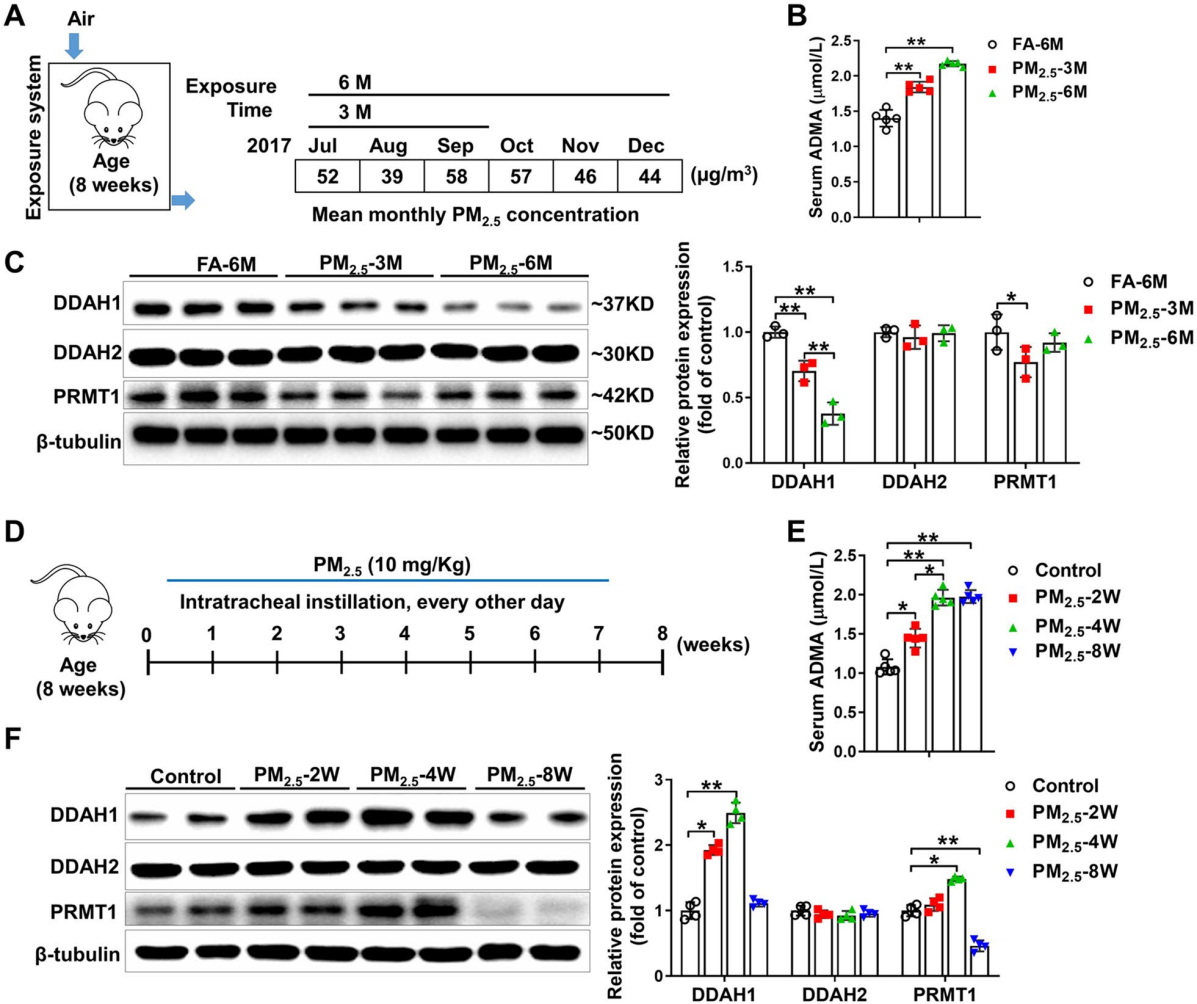
PM_2.5_ exposure affects the ADMA/DDAH pathway. **A** Experimental schema for chronic PM_2.5_ exposure is illustrated in diagram. The mean monthly PM_2.5_ concentrations from July to December 2017 are shown. **B** Mice were placed in the PM_2.5_ exposure system. After exposure to PM_2.5_ for 3 or 6 months, their serum ADMA levels were measured. Mice placed in the chamber with filtered air (FA) for 6 months were used as control. **C** The protein expression of dimethylarginine dimethylaminohydrolase 1 (DDAH1), DDAH2 and protein arginine methyltransferase 1 (PRMT1) in lung lysates were examined by Western blots. **D** Experimental schema for acute PM_2.5_ exposure is illustrated in diagram. **E** C57BL/6 mice were exposed to PM_2.5_ (10 mg/kg) every other day via intratracheal instillation as the indicated times, and then their serum ADMA levels were measured. Mice treated with PBS for 8 weeks were used as control. **F** Lysates of the lung tissue were examined by Western blots. In Fig. **B** and **E**, N = 5; in Fig. C, N = 3; in Fig. **F**, N = 4; Data are presented as the mean ± SD; *indicates p < 0.05, **indicates p < 0.01

*PM*_*2.5*_* exposure causes alterations in the ADMA/DDAH pathway*. To determine the effect of “real-world” PM_2.5_ exposure on the ADMA/DDAH pathway, we exposed the mice to ambient PM_2.5_ using the whole-body PM_2.5_ exposure system for 3 months or 6 months (July-December 2017), and the experimental processes are illustrated in Additional file 1: Figure S1A. As described previously [8, 10], the PM_2.5_ exposure system was located in the Zhongguancun campus of University of Chinese academy of Science, which is ~ 50 m away from a main traffic artery (Sihuan Road, Beijing, China). During the exposure period, PM_2.5_ was continuously collected using high-volume sampler particle collectors in the same place. The morphology of PM_2.5_ were examined by scanning electron microscopy. Dynamic light scattering measurement showed that the size range of collected PM_2.5_ was about 250–2500 nm and the mean size was 970.96 nm (Additional file 1: Figure S1). The concentrations of metals, soluble inorganic ions, polycyclic aromatic hydrocarbons (PAHs) and carbon in PM_2.5_ are listed in Additional file 2: Table S1. In addition, ambient air was pumped into the PM_2.5_ chamber and all PM with an aerodynamic diameter greater than 2.5 μM was removed by the swirler device. In the FA chamber, the pumped air was filtered with a high-efficiency particulate air filter. After PM_2.5_ exposure, serum ADMA levels were significantly increased. Mice in the PM_2.5_-6 M group had higher serum ADMA levels than mice in the PM_2.5_-3 M group (Fig. 1B). PM_2.5_ exposure decreased pulmonary DDAH1 expression in a time-dependent manner, but had no obvious effect on pulmonary DDAH2 expression. After 3 months of exposure to PM_2.5_, a significant reduction in pulmonary protein arginine methyltransferase 1 (PRMT1) expression was observed. However, ambient PM_2.5_ exposure for 6 months had no obvious effect on pulmonary PRMT1 expression (Fig. 1C). There were no significant differences in lung weight and the ratio of lung weight to bodyweight among the FA-6 M, PM_2.5_-3 M and PM_2.5_-6 M groups (Additional file 2: Table S2).

To investigate the effect of high concentration of PM_2.5_ exposure under some extreme conditions on ADMA/DDAH1 pathway, mice were subjected to acute PM_2.5_ exposure via intratracheal instillation for different time. The experimental protocol is illustrated in Fig. 1D and the dose used here was equal to daily exposure to ~ 1500 μg/m^3^ of PM_2.5_ [18]. As shown in Fig. 1E, serum ADMA levels were increased in PM_2.5_-exposed mice and PM_2.5_ exposure caused more increases in serum ADMA levels in the PM_2.5_-4 W group than in the PM_2.5_-2 W group. However, there was no significant difference in serum ADMA levels between the PM_2.5_-4 W and PM_2.5_-8 W groups (Fig. 1E). In addition, DDAH1 expression was increased in the lungs of mice from the PM_2.5_-2 W and PM_2.5_-4 W groups. However, the upregulation of pulmonary DDAH1 was diminished in the mice from the PM_2.5_-8 W group (Fig. 1F). DDAH2 expression was not affected by PM_2.5_ exposure in any group. In the mice from the PM_2.5_-4 W group, pulmonary PRMT1 was upregulated but it was dramatically downregulated in the mice from the PM_2.5_-8 W group (Fig. 1F). The lung weight and ratio of lung weight to bodyweight were significantly increased in acute PM_2.5_-exposed mice (Additional file 2: Table S3).

**Fig. 2:**
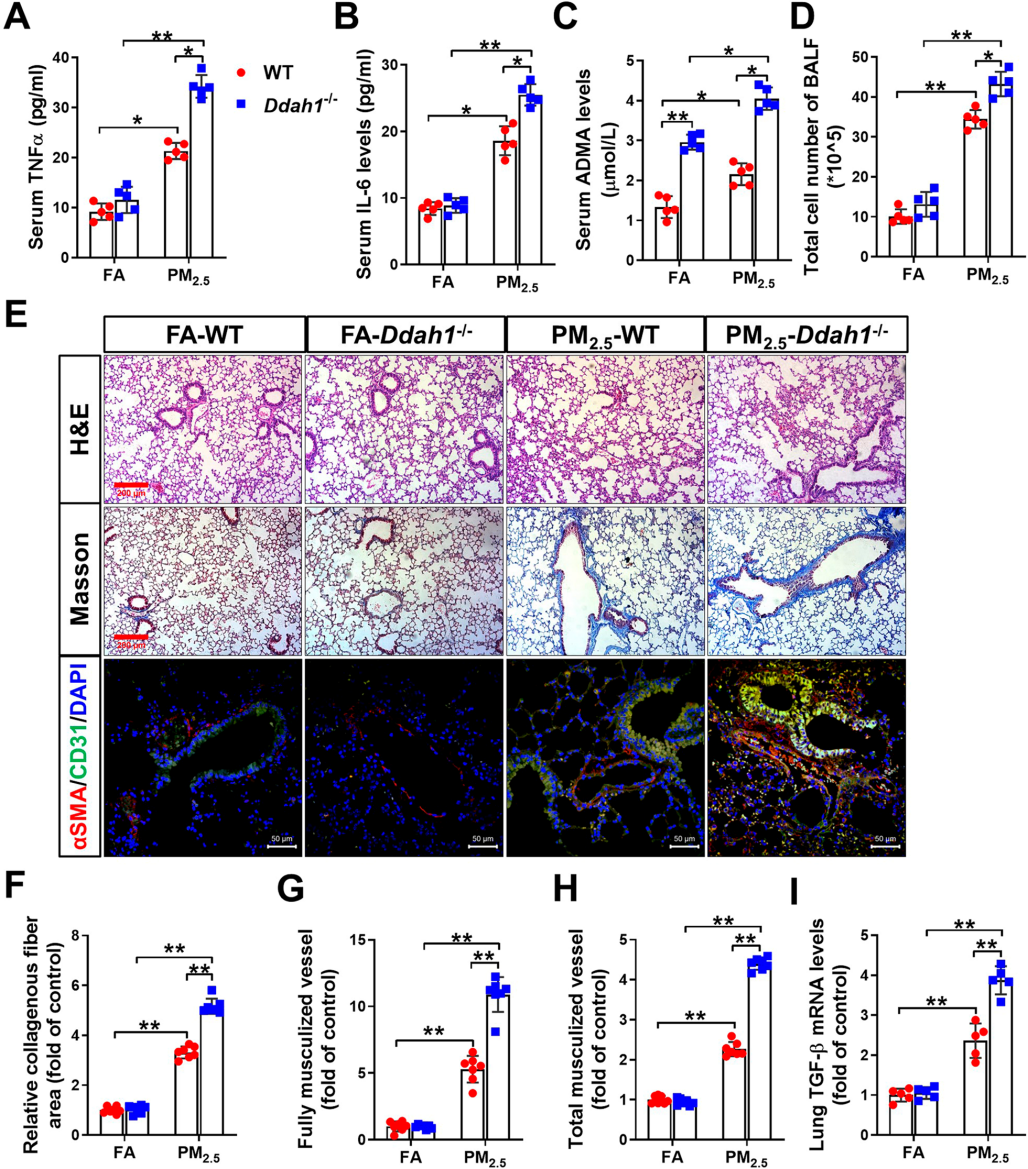
*Ddah1* deficiency exacerbates long-term PM_2.5_ exposure-induced systemic inflammation and lung injury. After 6 months of exposure to PM_2.5_, serum tumor necrosis factor alpha (TNFα) (**A**), interleukin 6 (IL-6) (**B**) and ADMA levels (**C**), as well as the cell numbers in the bronchoalveolar lavage fluid (BALF) (**D**), were measured, respectively. (**E**) Representative images of lung sections from FA- or PM_2.5_-exposed WT and *Ddah1*^−/−^ mice were stained with hematoxylin and eosin (H&E, Scale bar = 200 μm; the blue arrows point to widening of alveolar spaces, the green arrows point to inflammatory cell infiltration, the red arrows point to alveolar structure collapse), Masson’s trichrome stain (Scale bar = 200 μm; the deep red arrows point to collagen deposition), and antibodies specific for αSMA (green) and CD31 (red) (Scale bar = 50 μm). The relative collagenous fiber area (**F**), full and total muscularized vessels (**G**, **H**) and mRNA levels of lung TGF-β were quantified. In Figure **A**–**D** and **I**, N = 5; in Figure** F**–**H**, N = 7. Data are presented as the mean ± SD; *indicates p < 0.05, **indicates p < 0.01

Ddah1 deficiency exacerbates long-term PM_2.5_-induced systemic inflammation, lung vessel remodeling and fibrosis. To determine whether DDAH1 affects PM_2.5_-induced lung injury, we exposed WT and *Ddah1*^−/−^ mice to either ambient PM_2.5_ or FA for 6 months. During PM_2.5_ exposure, the body weights of the WT and *Ddah1*^−/−^ mice were recorded weekly. There was no obvious difference in the body weight changes between the WT and *Ddah1*^−/−^ mice (Additional file 1: Figure S2A). At the end of the experiment, there were still no significant differences in body weight, lung weight or the ratio of lung to body weight between the WT and *Ddah1*^−/−^ mice (Additional file 2: Table S4). After 6 months of PM_2.5_ exposure, the levels of tumor necrosis factor alpha (TNFα), interleukin 6 (IL-6) and ADMA in serum, as well as the number of cells in the bronchoalveolar lavage fluid (BALF), were significantly increased in both WT and *Ddah1*^−/−^ mice. However, these alternations were greater in *Ddah1*^−/−^ mice than in WT mice (Fig. 2A–D).

In addition, H&E staining of the lung sections revealed that chronic PM_2.5_ exposure resulted in obvious interstitial lung pathologic changes, as indicated by the widening of alveolar spaces (blue arrows), inflammatory cell infiltration (green arrows), and alveolar structure collapse (red arrows). Lung fibrosis is characterized by increased collagen deposition. As indicated by blue color area in Masson staining (deep red arrows), PM_2.5_ exposure caused lung fibrosis in both WT and *Ddah1*^−/−^ mice. Compared with the lungs of WT mice, lungs from *Ddah1*^−/−^ mice developed more severe morphological changes and fibrosis in response to PM_2.5_ (Fig. 2E, F). Vascular remodeling is a dynamic process that occurs in response to a variety of stimuli. In this study, vascular remodeling was determined histologically by staining for α-smooth muscle actin (αSMA) and CD31. The vessels in which > 75% of the vessel ring was encircled by smooth muscle cells are defined as fully muscularized vessels and vessels with 25% to 75% of the vessel ring encircled by smooth muscle cells were defined as partially muscularized vessels. As shown in Fig. 2E, PM_2.5_ exposure produced a greater number of muscularized vessels (including both fully and total muscularized vessels) in the *Ddah1*^−/−^ lungs than in WT lungs (Fig. 2E, G–H). PM_2.5_ exposure significantly increased TGF-β mRNA levels in the lungs of mice of both genotypes; however, this change was significantly greater in the lungs of *Ddah1*^−/−^ mice (Fig. 2I).

**Fig. 3:**
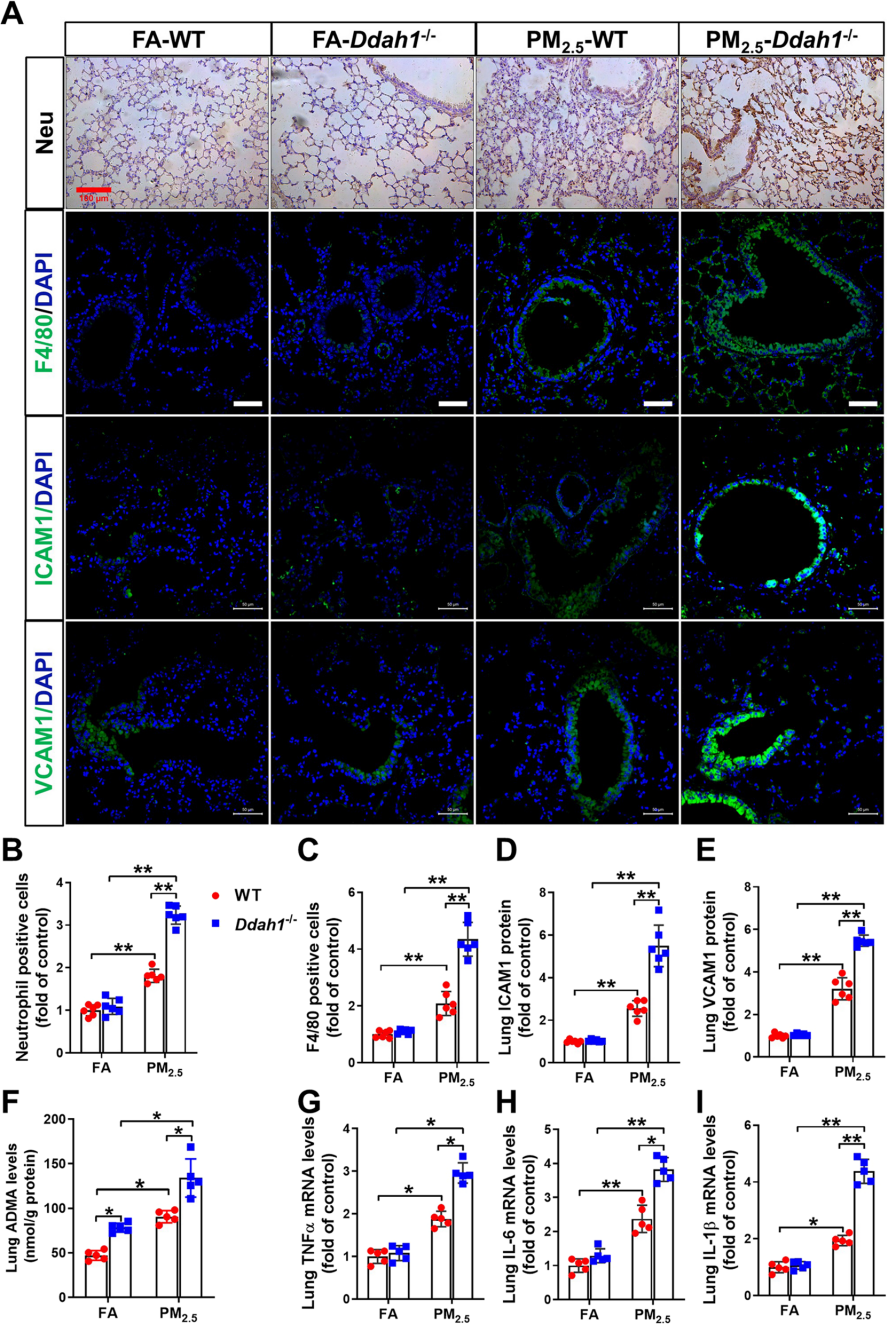
*Ddah1* deficiency aggravates lung inflammation in PM_2.5_-exposed mice. **A** Representative lung sections were stained with antibodies specific for neutrophils (Scale bar = 100 μm), F4/80, ICAM-1 and VCAM-1 (Scale bar = 50 μm). The relative neutrophil **B** or F4/80 **C** positive cell numbers and the levels of ICAM-1 (**D**) and VCAM-1 **E** were quantified. **F** The ADMA levels in lung tissue were measure. **G**–**I** The mRNA expression levels of TNFα, IL-6 and IL-1β in the lung tissues were measured. In Figure **B**–**E**, N = 6; In Figure **F**–**I**, N = 5; data are presented as the mean ± SD; *indicates p < 0.05, **indicates p < 0.01

*Ddah1 deficiency aggravates the pulmonary inflammatory response, cell death and oxidative stress in PM*_*2.5*_*-exposed mice*. The results of the immunohistochemical staining using antibodies against neutrophils and F4/80 (a macrophage-specific marker) showed that PM_2.5_ exposure resulted in serious infiltration of neutrophils and macrophages in *Ddah1*^−/−^ lungs compared to WT lungs (Fig. 3A–C). Vascular cell adhesion molecule 1 (VCAM-1) and intercellular cell adhesion molecule-1 (ICAM-1) contribute to inflammatory cells infiltration under various inflammatory conditions [19, 20]. Here, the pulmonary VCAM-1 and ICAM-1 distribution and expression in each experimental group were further examined by immune staining. *Ddah1* deficiency did not affect lung VCAM-1 and ICAM-1 levels in FA-exposed mice. PM_2.5_ exposure caused significant increases in lung VCAM-1 and ICAM-1 expression in both genotypes; however, these increases were significantly greater in the lung of *Ddah1*^−/−^ mice (Fig. 3A, D, E). *Ddah1* deficiency increased lung ADMA levels in both FA- and PM_2.5_-exposed mice (Fig. 3F). To determine whether PM_2.5_ exposure differentially stimulates pulmonary inflammatory responses in the lungs of WT and *Ddah1*^−/−^ mice, we performed qPCR to examine the mRNA levels of TNFα, IL-6 and IL-1β. PM_2.5_ exposure significantly increased the TNFα, IL-6 and IL-1β mRNA levels in the lungs of *Ddah1*^−/−^ mice compared to the lungs of WT mice (Fig. 3G–I).

**Fig. 4:**
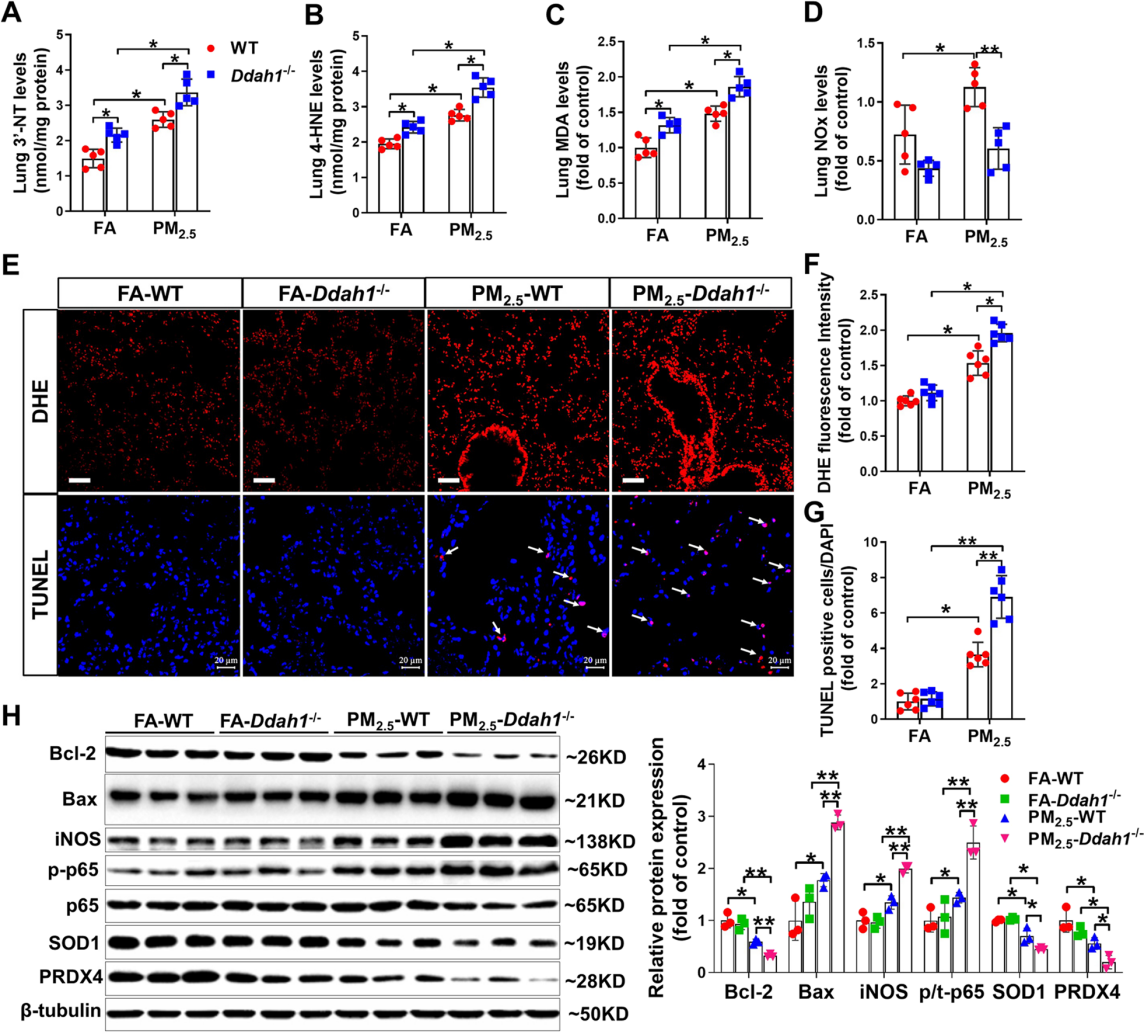
*Ddah1* deficiency exacerbates PM_2.5_-induced pulmonary oxidative stress and cell death. **A**–**D** After exposure to FA or PM_2.5_ for 6 months, 3′-nitrotyrosine (3′-NT) (**A**), 4-hydroxynonenal (4-HNE) (**B**), malondialdehyde (MDA) (**C**) and nitrate/nitrite (NOx) (D) levels in the lung tissue were measured. **E** Representative lung sections from FA- and PM_2.5_-exposed mice were stained with dihydroethidium (DHE) and a TUNEL assay kit (red) and DAPI (blue) (Scale bar = 50 μm, white arrows point to TUNEL-positive cells). **F**, **G** The relative fluorescence intensity of DHE (**F**) and the TUNEL-positive cells **G** were quantified. **H** Lysates of the lung tissue were examined by Western blots. β-tubulin was used as a loading control. In Figure **A**–**D**, N = 5; in Figure **F**–**G**, N = 6; in Figure **H**, N = 3; Data are presented as the mean ± SD; *indicates p < 0.05, **indicates p < 0.01

To determine whether *Ddah1* deficiency affects PM_2.5_-induced pulmonary oxidative stress, the changes in oxidative stress markers were measured. In the lungs of FA-exposed mice, *Ddah1* deficiency resulted in significant increase in 3′-nitrotyrosine (3′-NT), 4-hydroxynonenal (4-HNE) and malondialdehyde (MDA) levels (Fig. 4A–C). PM_2.5_ exposure increased the levels of 3′-NT, 4-HNE, and MDA in the lungs of WT and *Ddah1*^−/−^ mice; however, these changes were significantly greater in the lungs of *Ddah1*^−/−^ mice (Fig. 4A–C). Moreover, PM_2.5_ exposure also increased the pulmonary nitrate/nitrite (NOx) levels in WT mice and such increase was attenuated by *Ddah1* deficiency (Fig. 4D).

Next, we performed dihydroethidium (DHE) and TUNEL staining to assess the effect of *Ddah1* deficiency on superoxide generation and cell death. As shown in Fig. 4E, PM_2.5_ exposure resulted in higher superoxide levels and more TUNEL-positive cells in the lungs of *Ddah1*^−/−^ mice than in the lungs of WT mice (Fig. 4E–G). To explore the underlying mechanism by which DDAH1 affects PM_2.5_-induced lung injury, inflammation and cell death, we performed Western blotting to evaluate the expression levels of related proteins. Consistent with the TUNEL staining results, the expression of the pulmonary anti-apoptotic protein Bcl-2 was decreased, whereas the expression of the pro-apoptotic protein Bax was increased in PM_2.5_-exposed mice. These changes were more pronounced in *Ddah1*^−/−^ mice. In addition, significant increases in iNOS expression and the ratio of p-p65 (Ser536) to total p65 in the lungs of PM_2.5_-exposed mice were observed, and these increases were further exacerbated by *Ddah1* deficiency (Fig. 4H). Also, PM_2.5_ exposure significantly decreased the protein expression of SOD1 and PRDX4 in the lungs of WT mice, and these reductions were further exacerbated by *Ddah1* deletion (Fig. 4H).

**Fig. 5:**
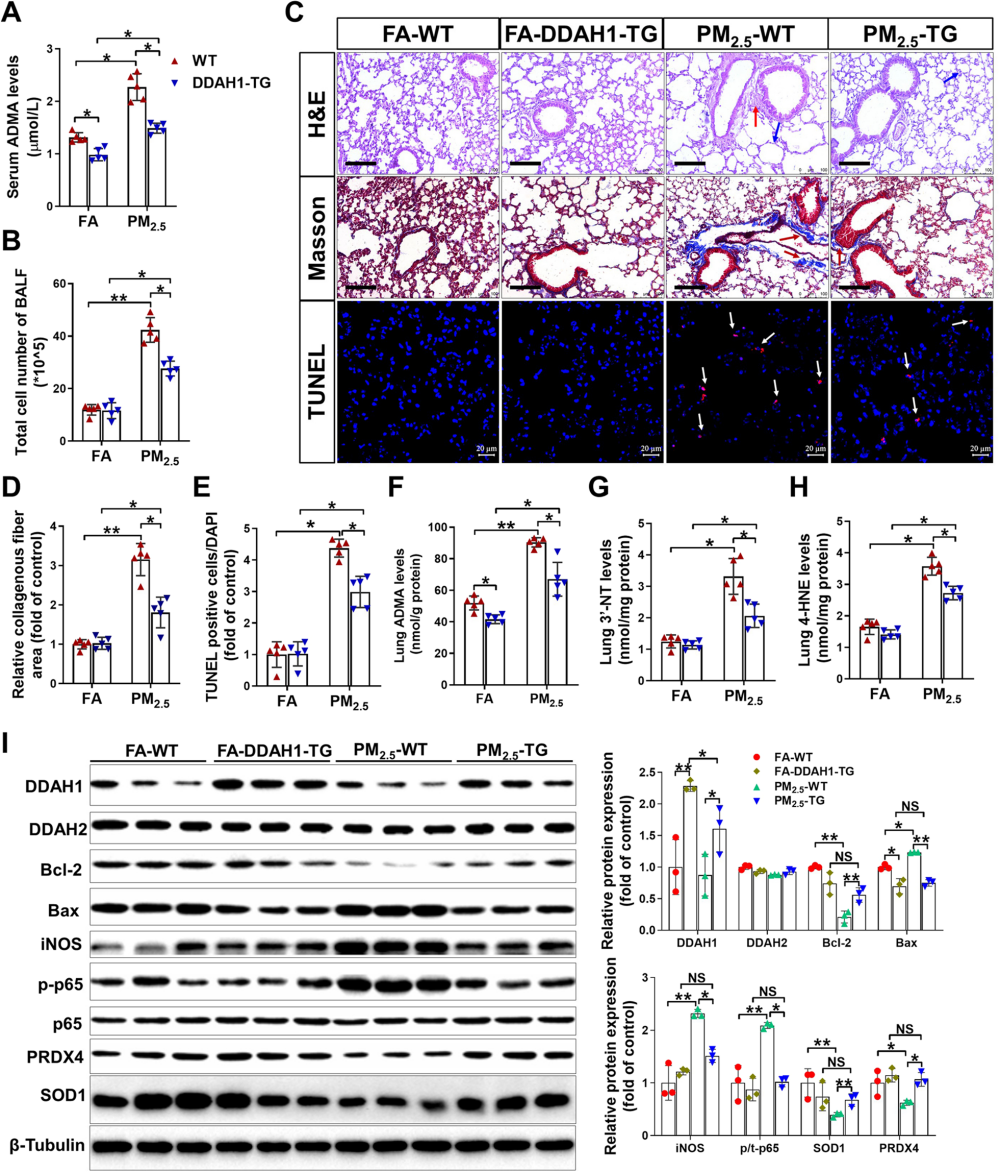
DDAH1 overexpression alleviates long-term PM_2.5_ exposure-induced pulmonary alveoli injury, fibrosis and inflammatory cell infiltration. After exposure for 6 months, serum ADMA levels (**A**) and cell numbers in BALF **B** were measured. **C** Representative lung sections from FA- or PM_2.5_-exposed WT and DDAH1-TG mice were stained with H&E (Scale bar = 100 μm; the blue arrows point to enlarged alveolar, the red arrows point to collapsed alveolar structure), Masson trichrome (Scale bar = 100 μm), and TUNEL kits (white arrows point to TUNEL positive cells, Scale bar = 20 μm). The relative collagenous fiber area (**D**) and TUNEL positive cell number **E** were quantified. In the lungs of FA- and PM_2.5_-exposed mice, ADMA (**F**), 3′-NT **G** and 4-HNE **H** were measured. **I** Lysates of lung tissue were examined by western blotting analysis. In Figure **A**–**H**, N = 5; in Figure **I**, N = 3; data are presented as the mean ± SD; *indicates p < 0.05, **indicates p < 0.01

*DDAH1 overexpression attenuates long-term PM*_*2.5*_*-induced lung injury, fibrosis and oxidative stress*. During the exposed period, the body weight of WT and DDAH1-TG mice was almost identical (Additional file 1: Figure S2B), and there were also no significant differences in body weight, lung weight and the ratio of lung to body weight at the end of the exposure period (Additional file 2: Table S5). After PM_2.5_ exposure, DDAH1-TG mice exhibited significantly less serum ADMA levels and cell influx in the BALF than WT mice (Fig. 5A, B). H&E and Masson’s staining revealed that the lungs of DDAH1-TG mice exhibited less morphological changes (indicated by blue and red arrows) in alveoli structure and collagen deposition (deep red arrows) than the lungs of the WT mice after PM_2.5_ exposure (Fig. 5C, D). In addition, TUNEL staining showed that PM_2.5_ exposure caused less TUNEL-positive cells in the lungs of DDAH1-TG mice than in the lungs of the WT mice (Fig. 5C, E). PM_2.5_ exposure caused significant increases in ADMA, 3′-NT and 4-HNE in the lungs of WT and DDAH1-TG mice; however, the increases in pulmonary 3′-NT, 4-HNE and MDA levels were significantly attenuated by DDAH1 overexpression (Fig. 5F–H). As shown in Fig. 5I, pulmonary DDAH1 expression in DDAH1-TG mice was ~ twofold higher than in WT mice, and was significantly decreased after PM_2.5_ exposure. DDAH2 expression was not affected by DDAH1 overexpression or PM_2.5_ exposure. In the lungs of PM_2.5_-exposed WT mice, Bcl-2, PRDX4 and SOD1 expressions were decreased whereas Bax and iNOS expression, as well as the p-p65(ser536) to t-p65 ratio were increased. However, downregulation of Bcl-2, PRDX4 and SOD1 and upregulation of iNOS, Bax and p-p65 ^ser536^ were significantly attenuated by DDAH1 overexpression (Fig. 5I).

**Fig. 6:**
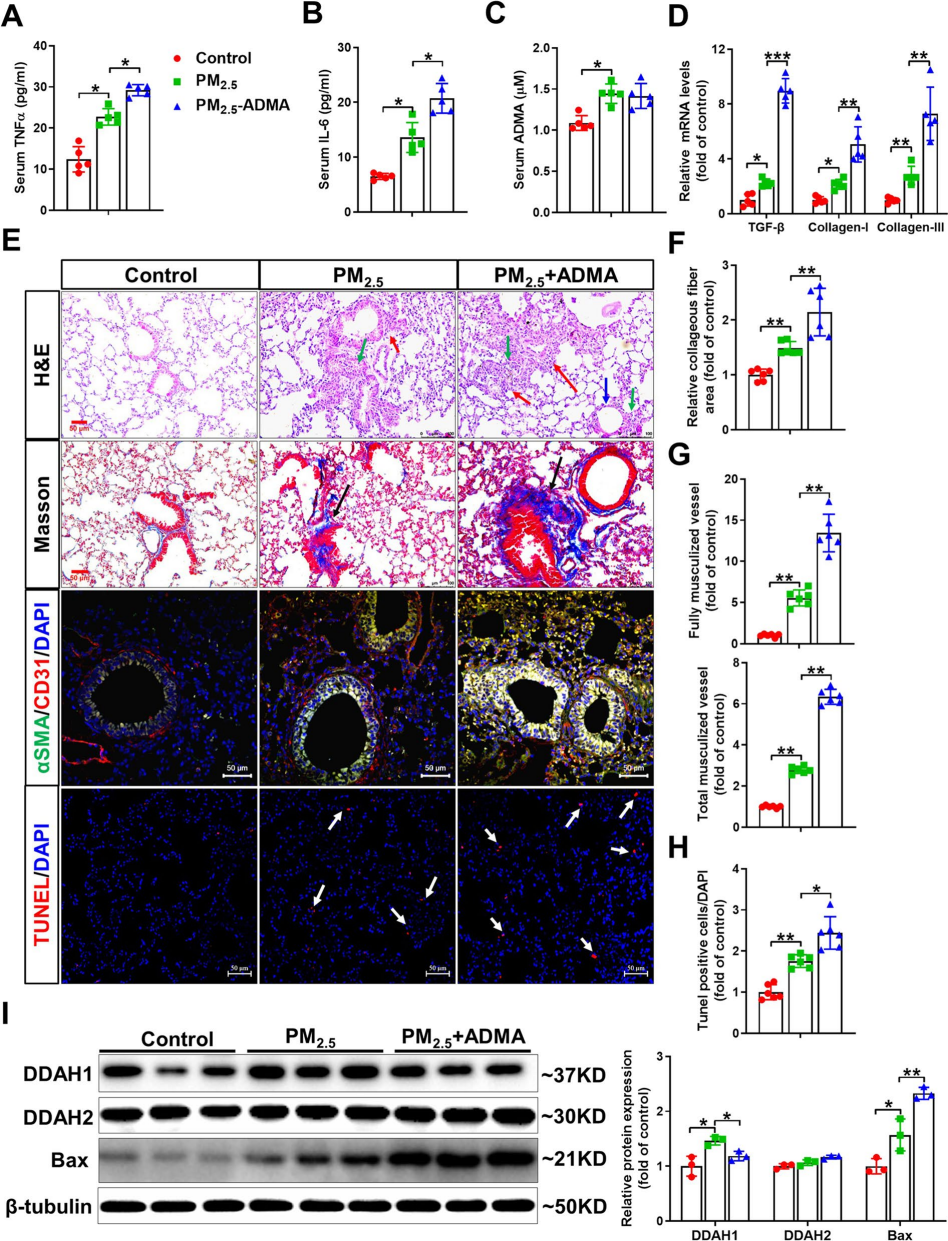
ADMA treatment exacerbates PM_2.5_-induced systemic inflammation, lung fibrosis, vessel remodeling and cell death. Mice were exposed to PM_2.5_ (10 mg/kg) with or without ADMA (2 mg/kg) every other day via intratracheal instillation for 2 weeks. **A**–**D** Serum TNFα (**A**), IL-6 (**B**) and ADMA **C** levels and mRNA levels of TGF-β and collagen I and III **D** were measured. **E** Representative lung sections were stained with H&E (the blue arrows point to widening of alveolar spaces, the green arrows point to inflammatory cell infiltration, the red arrows point to alveolar structure collapse), Masson’s trichrome kit (the black arrows point to collagen deposition), antibodies specific for αSMA (green) and CD31 (red), and TUNEL kit (red). Scale bar = 50 μm. **F**–**H** The relative collagenous fiber area (blue color area), muscularized vessels and TUNEL-positive cells (red point) were quantified. **I** Lung lysates were examined by Western blot. In Figure. **A**–**D**, N = 5; in Figure. F–H, N = 6; in Figure. **I**, N = 3; data are presented as the mean ± SD; * indicates p < 0.05, ** indicates p < 0.01

*ADMA treatment exacerbates PM*_*2.5*_*-induced systemic inflammation, lung vessel remodeling, fibrosis and cell death*. Next, we treated acute PM_2.5_-exposed mice with exogenous ADMA (2 mg/kg, every other day) for 2 weeks via intratracheal instillation to determine whether ADMA affects PM_2.5_-induced lung injury. PM_2.5_ exposure resulted in significant increases in the serum TNFα and IL-6 levels, and these increases were further enhanced by ADMA administration (Fig. 6A, B). Interestingly, ADMA treatment did not further increase serum ADMA levels in PM_2.5_-exposed mice (Fig. 6C). ADMA treatment significantly exacerbated the PM_2.5_-induced upregulation of TGF-β and collagen I and III (Fig. 6D). H&E staining of the lung sections showed that PM_2.5_-induced alveolar collapse (red arrows), inflammatory cell infiltration (green arrows) and airway epithelial thickening (blue arrows) were exacerbated by ADMA treatment (Fig. 6E). As shown by Masson’s trichrome staining, pulmonary collagen accumulation (black arrows) in PM_2.5_-exposed mice was further aggravated after ADMA treatment (Fig. 6E, F), indicating that ADMA could promote PM_2.5_-induced lung fibrosis.

After PM_2.5_ exposure, the fully muscularized vessels and total muscularized vessels (including both fully and partially muscularized vessels) were significantly increased in the lungs of PM_2.5_-exposed mice. ADMA treatment further promoted lung vessel muscularization in PM_2.5_-exposed mice, as demonstrated by the increased number of muscularized vessels (Fig. 6E, G). Moreover, TUNEL staining revealed that ADMA significantly increased the number of apoptotic cells in the PM_2.5_-exposed lungs (Fig. 6E, H). Western blot analysis showed that the upregulation of pulmonary DDAH1 in PM_2.5_-exposed mice was attenuated by ADMA treatment, whereas DDAH2 expression was unaffected after PM_2.5_ exposure and/or ADMA treatment (Fig. 6I). Consistent with the TUNEL results, the upregulation of the proapoptotic protein Bax caused by PM_2.5_ exposure was exacerbated by ADMA treatment (Fg. 6I).

**Fig. 7:**
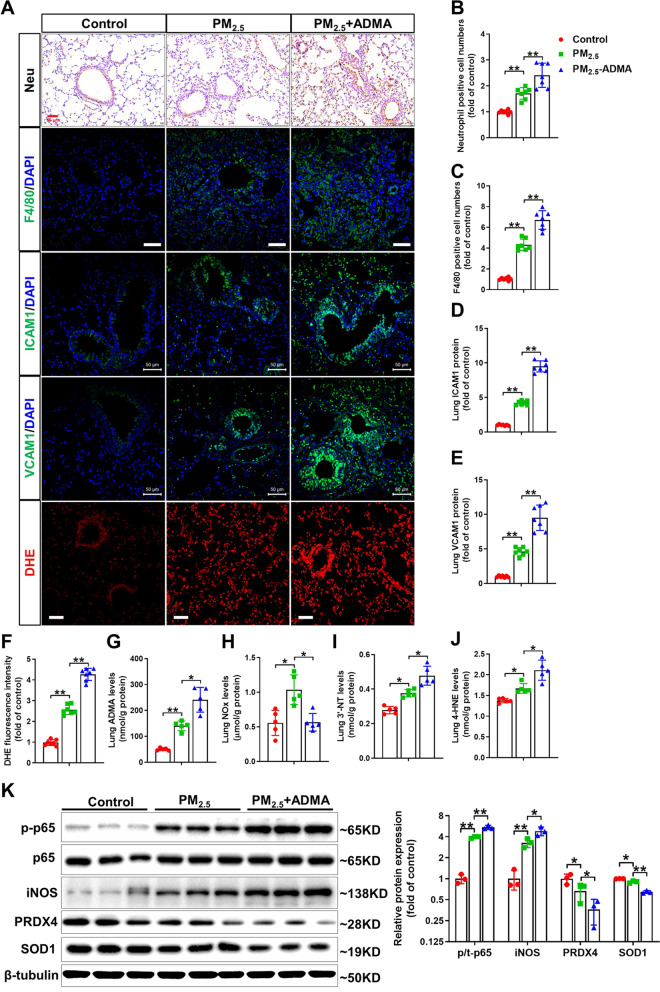
ADMA treatment promotes lung inflammation and oxidative stress in PM_2.5_-exposed mice. **A** Representative lung sections were stained with antibodies specific for neutrophils, F4/80, ICAM-1 and VCAM-1, and DHE. Scale bar = 50 μm. **B**–**F** The relative neutrophil- **B** or F4/80-positive **C** cell numbers, the levels of ICAM-1 (**D**) and VCAM-1 (**E**) and the relative fluorescence intensity of DHE **F** were quantified. **G**–**J** The ADMA (**G**), NOx (**H**), 3′-NT (**I**) and 4-HNE **J** levels in the lung tissue were measured. **K** Lung lysates were examined by Western blotting. In Fig. **B**–**J**, N = 5; in Fig. **K**, N = 3; data are presented as the mean ± SD; *indicates p < 0.05, **indicates p < 0.01

*ADMA treatment exacerbates pulmonary inflammation and oxidative stress in PM*_*2.5*_*-exposed mice*. Immunohistochemical staining showed that PM_2.5_ exposure increased infiltration of neutrophils and macrophages and expression of VCAM-1 and ICAM-1 in the lungs. Moreover, inflammatory cell infiltration and upregulation of VCAM-1 and ICAM-1 in the lungs of PM_2.5_-exposed mice were exacerbated by ADMA treatment (Fig. 7A–E). DHE staining demonstrated that pulmonary superoxide levels were increased in PM_2.5_-mice, while the increase was further enhanced by ADMA treatment (Fig. 7A, F). As expected, ADMA administration further increased ADMA levels but decreased NOx levels in PM_2.5_-exposed lungs (Fig. 7G, H). ADMA treatment also significantly increased pulmonary 3’-NT and 4-HNE levels in PM_2.5_-exposed mice (Fig. 7I, J). Western blot analysis revealed that PM_2.5_ exposure resulted in significant increases in phosphorylation of NF-κB p65 (Ser536) and iNOS expression, and these increases were enhanced by ADMA administration (Fig. 7K). ADMA treatment also aggravated the reduction in SOD1 and PRDX4 protein expression in PM_2.5_-exposed lungs (Fig. 7K).

**Fig. 8:**
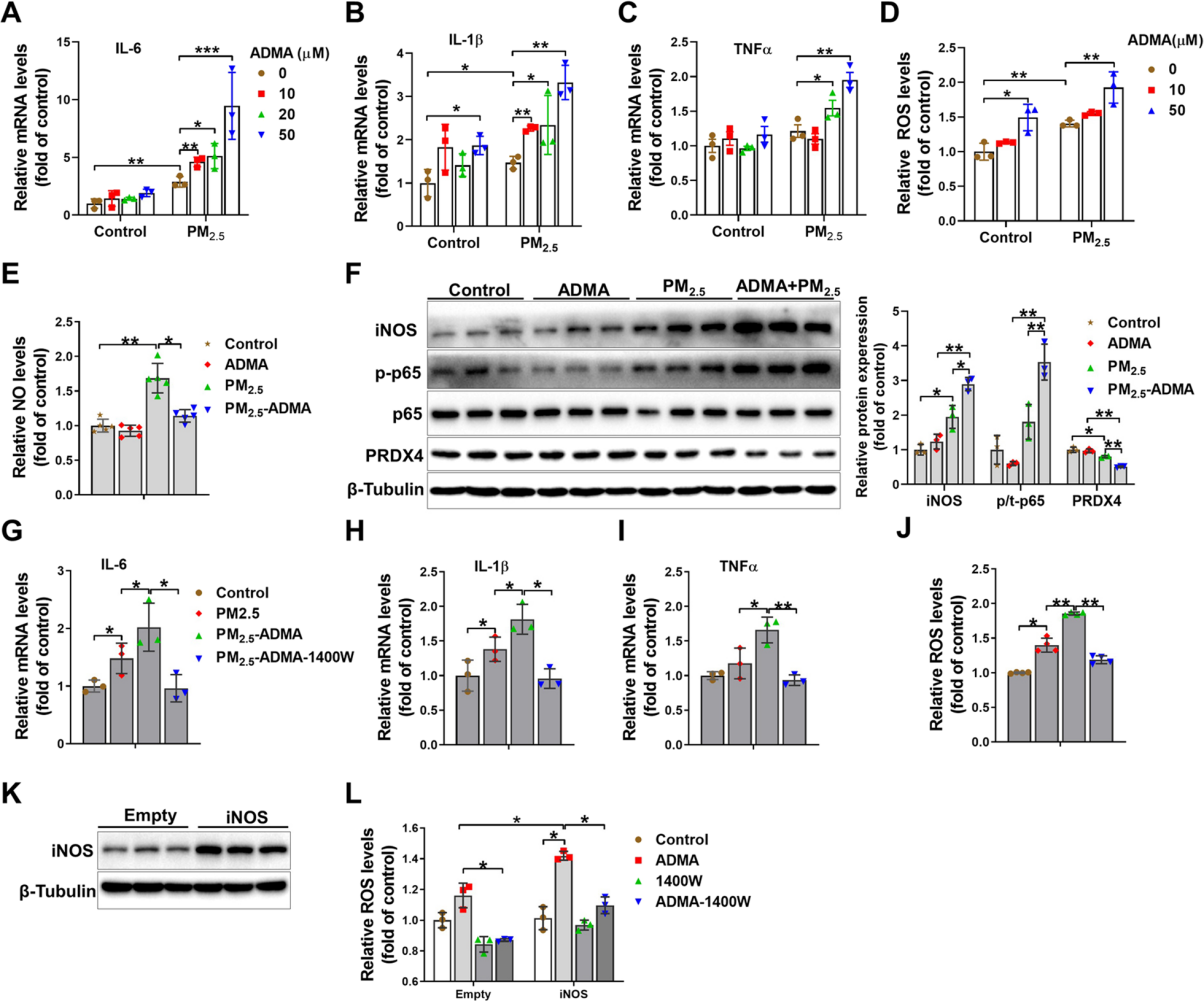
ADMA promotes the PM_2.5_-induced inflammatory response and oxidative stress in RAW264.7 macrophages. RAW264.7 macrophages were treated with 50 μg/ml PM_2.5_ with 0–50 μM ADMA for 6 h. **A**–**D** The mRNA levels of IL-6 (**A**), IL-1β (**B**) and TNFα (**C**) and the intracellular ROS levels **D** were measured. **E** Cells were treated with 50 μg/ml PM_2.5_ in the presence or absence of 50 μM ADMA for 6 h, and then intracellular NO levels were measured. **F** Cells were exposed to 50 μg/ml PM_2.5_ with or without 50 μM ADMA for 12 h, and then the cell lysates were examined by Western blot. **G**–**I** Cell were treated with 50 μg/ml PM_2.5_ with or without 20 μM ADMA or 2 μM 1400 W for 6 h, and then the mRNA levels of IL-6 (**G**), IL-1β (**H**) and TNFα (**I**) and the intracellular ROS levels (**I**) were measured. **K** Cells were transfected with PQCXIN-empty or PQCXIN-iNOS expression vector and cell lysates were examined by Western blot. **L** Control and iNOS-overexpressing cells were treated with 20 μM ADMA for 6 h, and then the intracellular ROS levels **I** were measured. N = 3, data are presented as the mean ± SD; *indicates p < 0.05, **indicates p < 0.01

*ADMA exacerbates the inflammatory response and ROS generation in PM*_*2.5*_*-exposed macrophages*. The induction of proinflammatory mediators by alveolar macrophages is a key factor in PM_2.5_-induced lung inflammation [21]. To determine whether ADMA affects the inflammatory response in PM_2.5_-exposed macrophages, RAW264.7 cells were exposed to 50 μg/ml PM_2.5_ in the presence or absence of ADMA for 6 h. Compared to the untreated control cells, treatment with 50 μM ADMA significantly increased the mRNA levels of IL-1β but had no obvious effect on the mRNA levels of IL-6 and TNFα. In PM_2.5_-exposed cells, ADMA treatment significantly increased the mRNA levels of IL-6, IL-1β and TNFα (Fig. 8A–C). Moreover, ADMA treatment also triggered more intracellular ROS generation in both control and PM_2.5_-exposed cells (Fig. 8D). PM_2.5_ exposure significantly increased intracellular NO levels, while the increase of NO was blocked by ADMA treatment (Fig. 8E). Western blot results showed that PM_2.5_ exposure increased iNOS and p-p65 expression and decreased PRDX4 expression in RAW264.7 cells, while these changes were further exacerbated by ADMA treatment (Fig. 8F). A previous report showed that ADMA can uncouple purified iNOS and cause superoxide generation [22]. To determine whether ADMA exacerbated PM_2.5_-induced the inflammatory response and oxidative stress by upregulating and/or uncoupling iNOS, cells were treated with the iNOS-specific inhibitor 1400 W, which caused significant decreases in the mRNA levels of IL-6, IL-1β and TNFα and intracellular ROS levels in PM_2.5_ plus ADMA-treated cells (Fig. 8G–J). We also transfected cells with PQCXIN–iNOS expression vector, which increased iNOS expression approximately threefold (Fig. 8K). Interestingly, ADMA caused more ROS generation in iNOS-overexpressing cells than in control cells, and this effect was diminished by 1400 W (Fig. 8L).

## Discussion

There are two new major findings in this study. First, we demonstrated that *Ddah1* deficiency exacerbated, whereas DDAH1 overexpression attenuated, long-term PM_2.5_ exposure-induced lung injury. Second, the protective role of DDAH1 in attenuating PM_2.5_-induced injury was associated with decreases in ADMA levels and the attenuation of inflammation and oxidative stress. Our data might provide novel insights into the pathogenesis of lung injury after PM_2.5_ exposure.

Epidemiological studies have consistently demonstrated that ADMA is an independent risk factor for cardiovascular and/or respiratory diseases [23–25]. Circulating levels of ADMA are elevated under several pathological conditions, including pulmonary hypertension [26], COPD [27], asthma [28] and congestive heart failure [29]. In mouse models, exposure to lipopolysaccharides (LPS) [30], ovalbumin [13] or cigarette smoke [31] also increases serum ADMA levels. ADMA accumulation under those pathological conditions may be associated with the downregulation of DDAH1 [32]. In the present study, we demonstrated that serum ADMA levels were also increased in PM_2.5_-exposed mice, which was accompanied by pulmonary DDAH1 reduction in long-term PM_2.5_-exposed mice. However, ADMA accumulation in acute PM_2.5_-induced mice was associated with the upregulation of PRMT1, but not the downregulation of DDAH1. These results implied that acute PM_2.5_ exposure increases ADMA levels by promoting its production, whereas long-term PM_2.5_ exposure causes ADMA accumulation by inhibiting its degradation.

Consistent with previous reports demonstrating that ADMA potentiates ovalbumin- or cigarette smoke-induced lung injury [14, 31], we found that PM_2.5_ exposure increased lung ADMA levels and that *Ddah1* deficiency or ADMA administration exacerbated PM_2.5_-induced lung fibrosis, vessel remodeling, inflammation and oxidative stress. In contrast, decreased lung ADMA levels by overexpressing DDAH1 ameliorated PM_2.5_-induced lung injury. These findings suggested that elevated lung ADMA levels play an important role in PM_2.5_-induced lung injury.

As an inhibitor of NOS, ADMA can induce endothelial dysfunction in cardiovascular disease by inhibiting NOS activity [23]. Consistently, we found that *Ddah1* deficiency or ADMA administration decreased lung NOx levels in PM_2.5_-exposed mice. Under certain stress conditions, ADMA accumulation can uncouple eNOS [33] or iNOS [22] to promote ROS production. Here, we found that *Ddah1* deficiency or ADMA administration significantly increased iNOS expression in PM_2.5_-exposed lungs, which was associated with elevated oxidative stress. ADMA also caused more increases in intracellular ROS levels in iNOS-overexpressing cells than in control macrophages. In addition, *Ddah1* deficiency or ADMA administration significantly decreased, while DDAH1 overexpression increased the expression of SOD1 and PRDX4 in PM_2.5_-exposed lungs, which may also contribute to PM_2.5_-induced oxidative stress [10]. Thus, it is likely that ADMA exacerbated PM_2.5_-induced lung injury by promoting ROS production in an iNOS-dependent manner and changing the antioxidant system in the airway. Notably, the effects of ADMA on ROS generation and cell death were dependent on the cell types and stresses applied to the cells. For example, ADMA promotes ROS production in LPS- or oxidized low-density lipoprotein-treated macrophages [34, 35]. However, ADMA does not affect intracellular ROS levels or tert-butyl hydroperoxide-induced cell death in mouse embryonic fibroblasts (MEFs) [36].

Overexpressing DDAH1 attenuates lung inflammation in mice exposed to ovalbumin, agricultural organic dust extract, house dust mites or cigarette smoke [14–16, 31]. Here, *Ddah1* deficiency or ADMA administration increased the serum levels of TNFα and IL-6, enhanced the infiltration of inflammatory cells and elevated the expression levels of inflammatory genes in PM_2.5_-exposed mice, suggesting that DDAH1 may protect against PM_2.5_-induced lung injury by repressing the inflammatory response. Moreover, the finding that *Ddah1* deficiency increased VCAM-1 and ICAM1 expression suggested that DDAH1 may attenuate lung inflammation by downregulating adhesion molecules, which contributes to the recruitment of circulating leukocytes to the lung in PM_2.5_-exposed mice [37].

As a pivotal mediator of inflammatory responses, NF-κB is activated in PM_2.5_-exposed epithelial cells [38] and lungs [39] and then induces the upregulation of various proinflammatory cytokines and chemokines. NF-κB also mediates the induction of iNOS expression by exogenous stimuli, and inhibition of iNOS could block NF-κB activation in PM_2.5_-exposed cells [38]. In the present study, we found that *Ddah1* deficiency exacerbated, whereas DDAH1 overexpression attenuated, NF-κB activation and iNOS induction in the lungs of PM_2.5_-exposed mice suggests a potential mechanism for the anti-inflammatory effect of DDAH1. Previous reports have reported that exogenous ADMA activates NF-κB in cigarette smoke-exposed lungs [31], while *Ddah1* deletion also activates NF-κB in MEFs in a ROS-dependent manner [36]. It is possible that DDAH1 represses NF-κB activity by degrading ADMA or reducing ROS production.

Recently, we have consistently demonstrated that DDAH1 exerts antioxidative effects in different cell models [36, 40], fatty livers [41], and aged and diabetic kidneys [42]. DDAH1 also plays an important role in attenuating monocrotaline-induced lung oxidative stress in the lungs of rats [43]. Consistent with these reports, we found that *Ddah1* deficiency exacerbated, whereas DDAH1 overexpression attenuated, PM_2.5_-induced increases in pulmonary 3’-NT, 4-HNE and MDA levels, indicating that DDAH1 also protects against PM_2.5_-induced lung injury, at least partially, by attenuating oxidative stress. Although PM_2.5_ has the ability to directly increase ROS levels [44], it may also promote oxidative stress by decreasing the expression of antioxidant enzymes [8, 10]. SOD1 as an antioxidant enzyme that scavenges superoxide in the cytoplasm, and PRDX4 scavenges H_2_O_2_ in the ER [45]. Thus, the finding that DDAH1 positively regulates SOD1 and PRDX4 expression indicates an important mechanism for the antioxidative effect of DDAH1 in the lungs of PM_2.5_-exposed mice. As DDAH1 was shown to regulate SOD2 in MEFs [36] and PRDX5 in tubular epithelial cells [42], it is likely that the regulation by DDAH1 of antioxidant enzymes is dependent on the tissue or cell type.

## Conclusions

In summary, our study suggests that DDAH1 protects against long-term PM_2.5_ exposure-induced lung injury damage by degrading ADMA and repressing p65 phosphorylation and iNOS induction, thereby decreasing oxidative stress and inflammation. Our results suggest that DDAH1 plays an important role in protecting lung against air pollution induced lung injury.

## Materials and methods

### Reagents

ADMA, 2,7-dichlorodihydrofluorescein diacetate (DCFH-DA), DHE and 1400 W were purchased from Sigma Chemical Co. (#D4268, #D6883, #D7008 and # W4262, St. Louis, MO, USA). BCA protein concentration, MDA, total NOx assay kits, TUNEL kits and DAF-FM DA were purchased from the Beyotime Institute of Biotechnology (#P0012, #S0131S, #S0023, #C1090 and #S0019, Shanghai, China). ADMA ELISA kit was obtained from BioVision Inc. (#E4521-100, Milpitas, CA, USA). ELISA kits for mouse TNFα, IL-6, 3′-NT and 4-HNE were purchased from Abcam PLC (#ab208348, #ab100712, #ab116691 and #ab238538, Cambridge, UK). The Masson’s trichrome staining kit was obtained from Solarbio Science & Technology Co. LTD (#G1340, Beijing, China).

### Animal experiments

The *Ddah1*^−/−^ mice were generated as previously reported [46]. In brief, the DDAH1^flox/flox^ mice were crossed with protamine (Prm)-Cre mice to delete the *Ddah1* gene in the sperm of the male Prm-Cre/DDAH1^flox/+^ mice. These male mice were crossed with WT female mice to obtain *Ddah1*^+/−^ mice, and these heterozygotes were continuously crossed with C57BL/6 mice for more than 10 generations. The homozygote *Ddah1*^−/−^ mice and WT littermates (12-week-old, male, 8–10 mice per group) used in this study were generated by inbreeding of the *Ddah1*^+/−^ heterozygotes. The DDAH1-TG mice (C57BL/6 background) were generated by Cyagen Biosciences Inc. (Jiangsu, China) using a human DDAH1 transgenic vector, which contains human DDAH1 coupled with 3xFLAG epitope on N-terminus, an EF1A promoter and RNA processing signals from SV40 polyA [47]. The DDAH1-TG mice and WT littermates (8-week-old, male, 8–10 mice per group) used in this study were obtained through crossing the heterozygote DDAH1-TG mice with C57BL/6 mice.

For chronic PM_2.5_ exposure, 8-week-old male C57BL/6 J mice were randomly divided into 3 groups (10 mice/group). Mice were placed in the whole-body PM_2.5_ exposure system for 3 or 6 months (defined as the PM_2.5_-3 M and PM_2.5_-6 M groups). Mice placed in the FA chamber for 6 months (defined as the FA-6 M group) were used as control. The *Ddah1*^−/−^, DDAH1-TG and their respective WT littermates were also placed in the FA and PM_2.5_ chamber for 6 months. Detail information on the PM_2.5_ exposure system has been described previously [8, 10, 48]. The individually ventilated cages were placed in the exposure system for 12 h/day, 7 days/week. Mice were fed commercial mouse chow and distilled water ad libitum during the whole exposure stage (2017/07/02–2017/12/31). The cages were taken out the chambers every 3 days for cleaning and replacing fresh food and water.

For acute PM_2.5_ exposure, 8- to 10-week-old male C57BL/6 J mice were randomly divided into 3 groups (10 mice/group). The mice were treated with 20 μl PBS or 10 mg/kg PM_2.5_ with or without 2 mg/kg ADMA in 20 μl PBS every other day for 2 weeks. To determine the effect of exposure time on the ADMA/DDAH pathway, 8-week-old male C57BL/6 J mice were also randomly divided into three groups (6 mice/group), and mice were exposed to 10 mg/kg PM_2.5_ every other day for 2, 4, or 8 weeks via intratracheal instillation (defined as the PM_2.5_-2 W, PM_2.5_-4 W and PM_2.5_-8 W groups).

### Bronchoalveolar lavage

After PM_2.5_ exposure, the mice were anesthetized with 1% pentobarbital sodium, and then their lungs were lavaged 3 times with 1 ml PBS. The BALF was collected and centrifuged at 1000 rpm for 10 min at 4 °C. The BAL cell pellets were resuspended in 100 μl PBS followed by counting the number of cells.

### Sample collection and biochemical assay

At the end of experiment, mice were euthanized using spinal cord dislocation method. The blood samples collected from orbital sinus were placed in the serum separator tubes and clotted for 30 min at room temperature. Then the tubes were centrifugated at 3000 rpm for 10 min to get serum for biochemical assay. Serum ADMA, TNFα and IL-6 levels were measures using the respective ELISA kits. The mice lungs were collected after saline perfusion. The lung NOx levels were determined by the total NOx assay kits.

### Histological assessment

As described previously [49], lung paraffin Sects. (5 μm) were stained with H&E, Masson’s trichrome staining kits, DHE, specific antibodies and TUNEL kits.

### Cell culture and treatment

The murine RAW 264.7 cell line was obtained from the China Infrastructure of Cell Line Resource (Beijing, China) and cultured in Dulbecco's modified Eagle’s medium supplemented with 10% fetal bovine serum and 1% penicillin and streptomycin at 37 °C with 5% CO_2_. After culturing for 24 h, the culture medium was replaced with serum-free medium and the cells were exposed to freshly dispersed PM_2.5_ preparations with 0–50 μM ADMA or 2 μM iNOS specific inhibitor (1400 W) for 6 or 12 h. The intercellular ROS and NO levels were measured by spectrophotometry using DCFH-DA and DAF-FM DA, respectively [17]. In brief, the untreated control and treated cells were washed with PBS and then incubated with 5 μM fluorescence dyes at 37 °C for 30 min. Then cells were washed three times and the fluorescence intensity was determined using a Synergy H1 Hybrid Multi-Mode Microplate Reader (BioTek Instruments, Inc., Winooski, VT, USA).

### Quantitative real-time PCR and Western blots

The detailed protocols for cDNA synthesis, quantitative real-time PCR, protein extraction and Western blots were described previously [49]. The primary antibodies and primers used in this study are listed in Additional file 2: Table S6 and Additional file 2: Table S7, respectively.

### Statistical analysis

All data are expressed as the mean ± standard deviation (SD). The significance of differences was evaluated using an unpaired 2-tailed *t* test or one-way or two-way ANOVA followed by Fisher’s least significant difference test using GraphPad Prism 8 (GraphPad Software Inc., CA, USA). Differences were considered at a *P* level of less than 0.05.

## Supplementary Information

**Additional file1**. **Fig. S1. **The morphology and size distribution of PM_2.5_.** A** Scanning electron microscopy image of PM_2.5_. Scale bar = 500 nm. **B** Particle size distribution in the ultrapure water was analyzed by dynamic light scattering. **Fig. S2. **The body mass curves of PM_2.5_-exposed mice. During the exposure period, body weight of *Ddah1*^−/−^ mice and wild type (WT) littermates (**A**), and body weight of human DDAH1 transgenic mice (DDAH1-Tg) and WT littermates were recorded every two weeks. N=8–10, data are presented as mean ± SD.

**Additional file2**. **Table S1**. Metals, soluble inorganic ions, polycyclic aromatic hydrocarbons (PAHs) and carbon in the PM_2.5_ samples. **Table S2**. Anatomic data for filter air and long-term PM_2.5_*-*exposed mice. **Table S3**. Anatomic data for control and acute PM_2.5_*-*exposed mice. **Table S4**. Anatomic data for wild type and *Ddah1*^−/−^ mice after PM_2.5_ exposure. **Table S5**. Anatomic data for wild type and DDAH1-TG mice after PM_2.5_ exposure. **Table S6**. Detail information for antibodies. **Table S7**. The quantitative real-time PCR primer information.

## Data Availability

The datasets during and/or analyzed during the current study are available from the corresponding author on reasonable request.

## Abbreviations

3′-NT: 3′-Nitrotyrosine
4-HNE: 4-Hydroxynonenal
MDA: Malondialdehyde
ADMA: Asymmetrical dimethylarginine
αSMA: Alpha smooth muscle actin
COPD: Chronic obstructive pulmonary disease
DDAH: Dimethylarginine dimethylaminohydrolase
l-Arg: l-Arginine
NO: Nitric oxide
DHE: Dihydroethidium
FA: Filtered air
H&E: Hematoxylin and eosin
ICAM-1: Intercellular cell adhesion molecule-1
IL: Interleukin
NOS: Nitric oxide synthase
NOx: Nitrate/nitrite
PRDX: Peroxiredoxin
PRMT1: Protein arginine methyltransferase 1
ROS: Reactive oxygen species
SOD: Superoxide dismutase
TNFα: Tumor necrosis factor alpha
VACM-1: Vascular cell adhesion molecule 1
