# Supplementary material for: Dimethylarginine dimethylaminohydrolase 1 protects PM2.5 exposure-induced lung injury in mice by repressing inflammation and oxidative stress
Source: Part Fibre Toxicol. 2022 Oct 14;19:64. doi: 10.1186/s12989-022-00505-7 (PMC9569114; doi:10.1186/s12989-022-00505-7)
Supplement: Supplementary file 1 — Additional file1. Fig. S1. The morphology and size distribution of PM2.5. A Scanning electron microscopy image of PM2.5. Scale bar = 500 nm. B Particle size distribution in the ultrapure water was analyzed by dynamic light scattering. Fig. S2. The body mass curves of PM2.5-exposed mice. During the exposure period, body weight of Ddah1−/− mice and wild type (WT) littermates (A), and body weight of human DDAH1 transgenic mice (DDAH1-Tg) and WT littermates were recorded every two weeks. N=8–10, data are presented as mean ± SD. [file 12989_2022_505_MOESM1_ESM.pptx]

## Slide 1
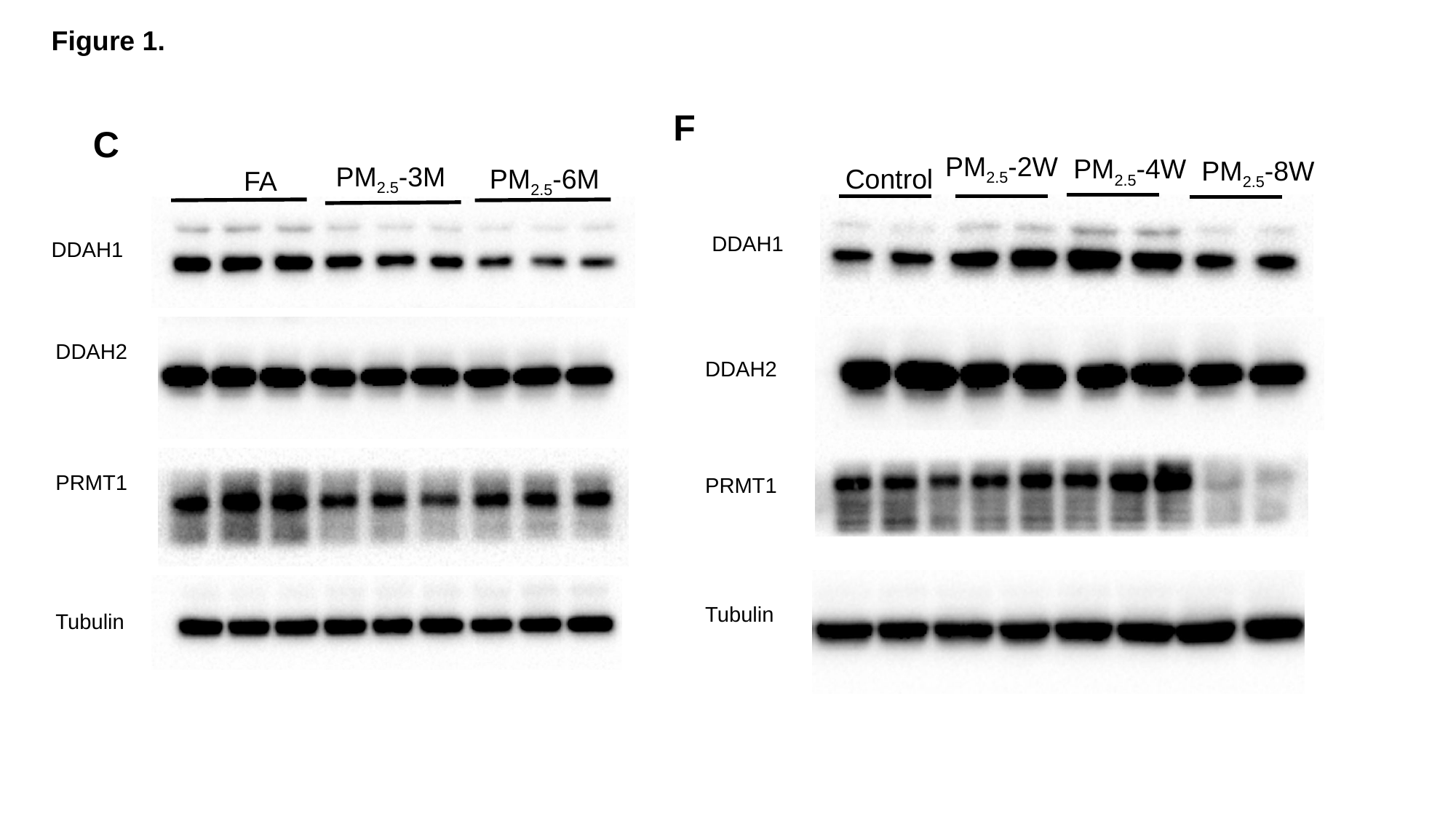

Figure 1.
F
C
PM2.5-2W
PM2.5-4W
PM2.5-8W
PM2.5-3M
PM2.5-6M
Control
FA
DDAH1
DDAH1
DDAH2
DDAH2
PRMT1
PRMT1
Tubulin
Tubulin

## Slide 2
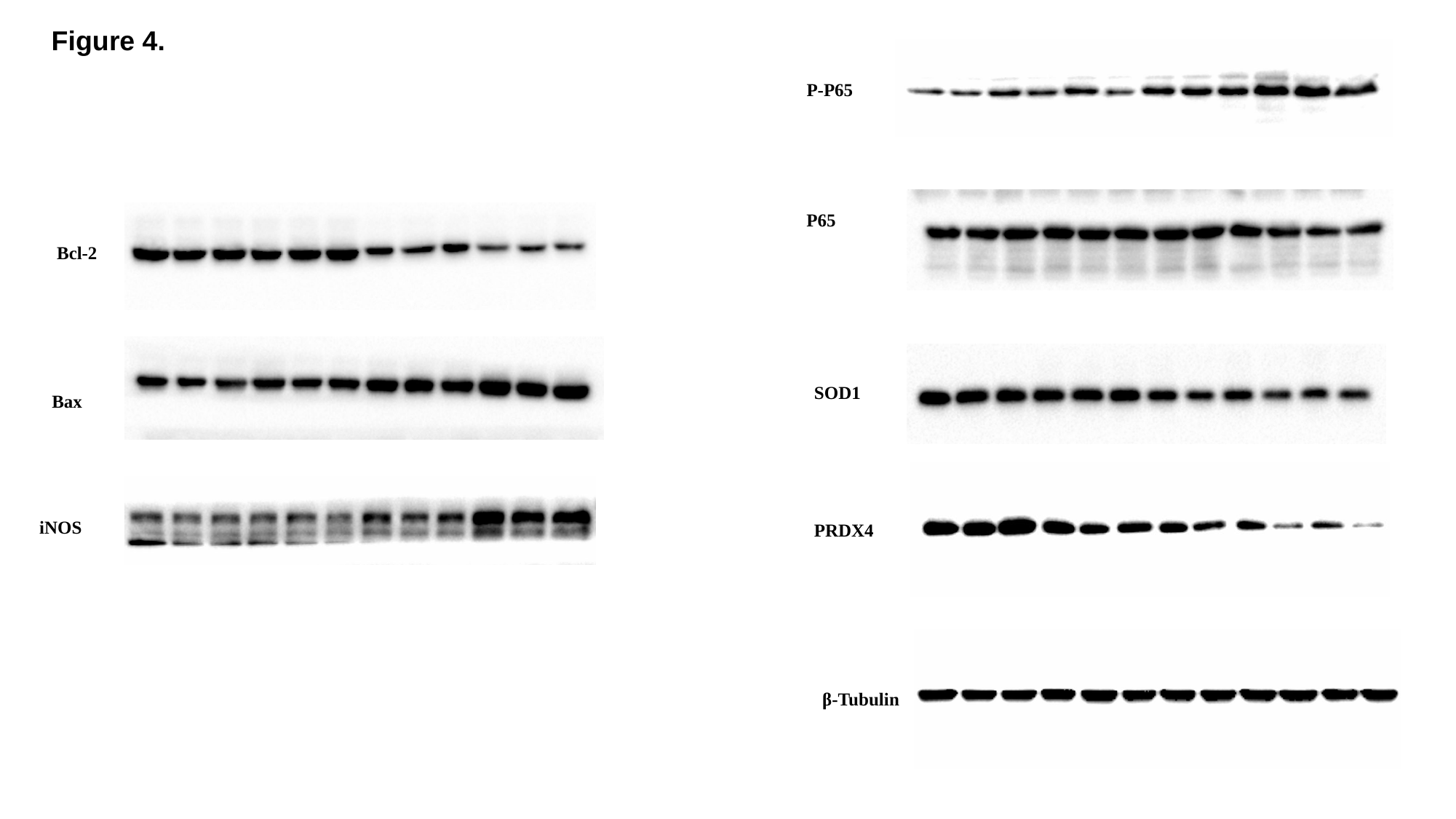

Figure 4.
P-P65
P65
Bcl-2
SOD1
Bax
iNOS
PRDX4
β-Tubulin

## Slide 3
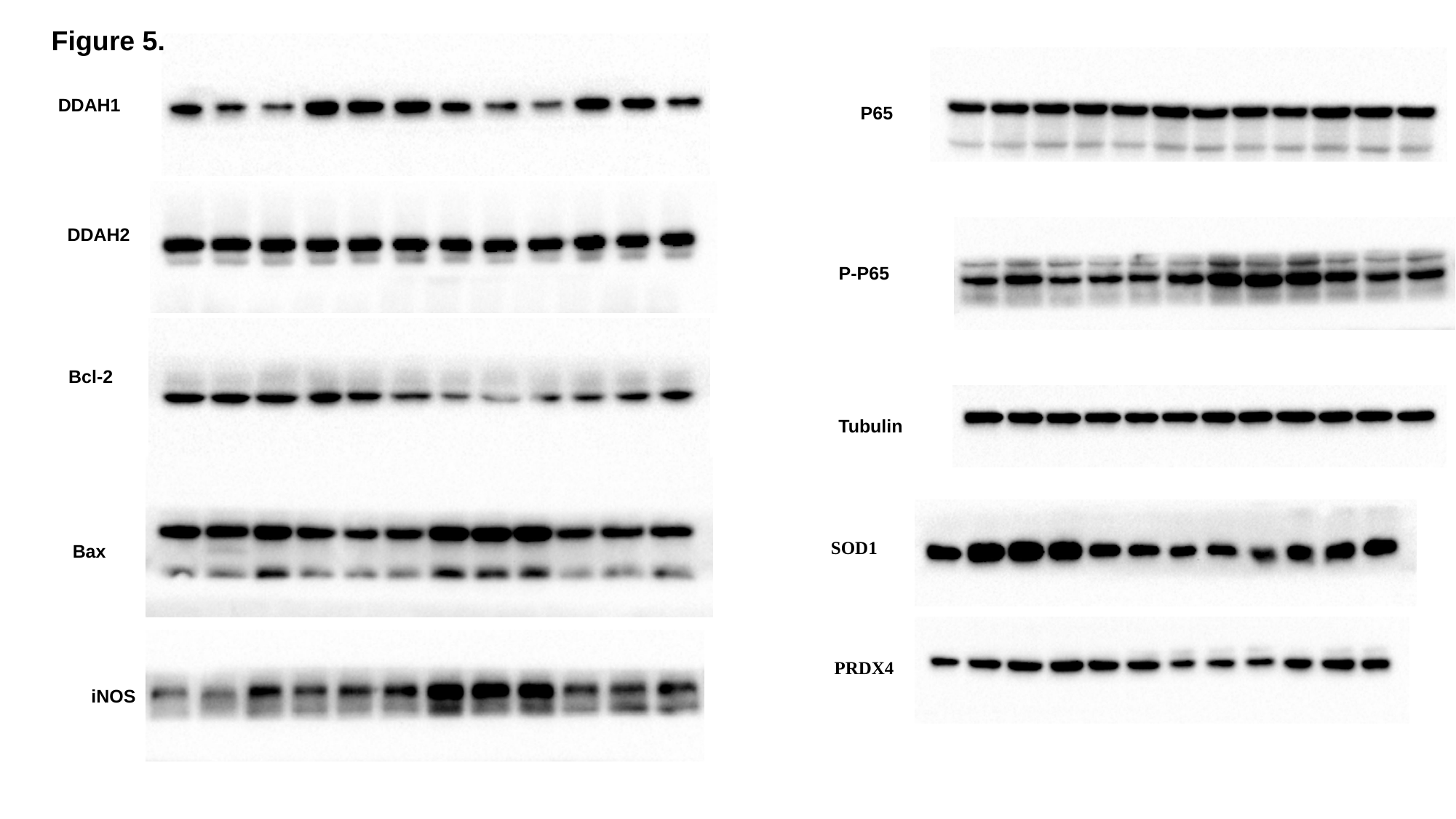

Figure 5.
DDAH1
P65
DDAH2
P-P65
Bcl-2
Tubulin
SOD1
Bax
PRDX4
iNOS

## Slide 4
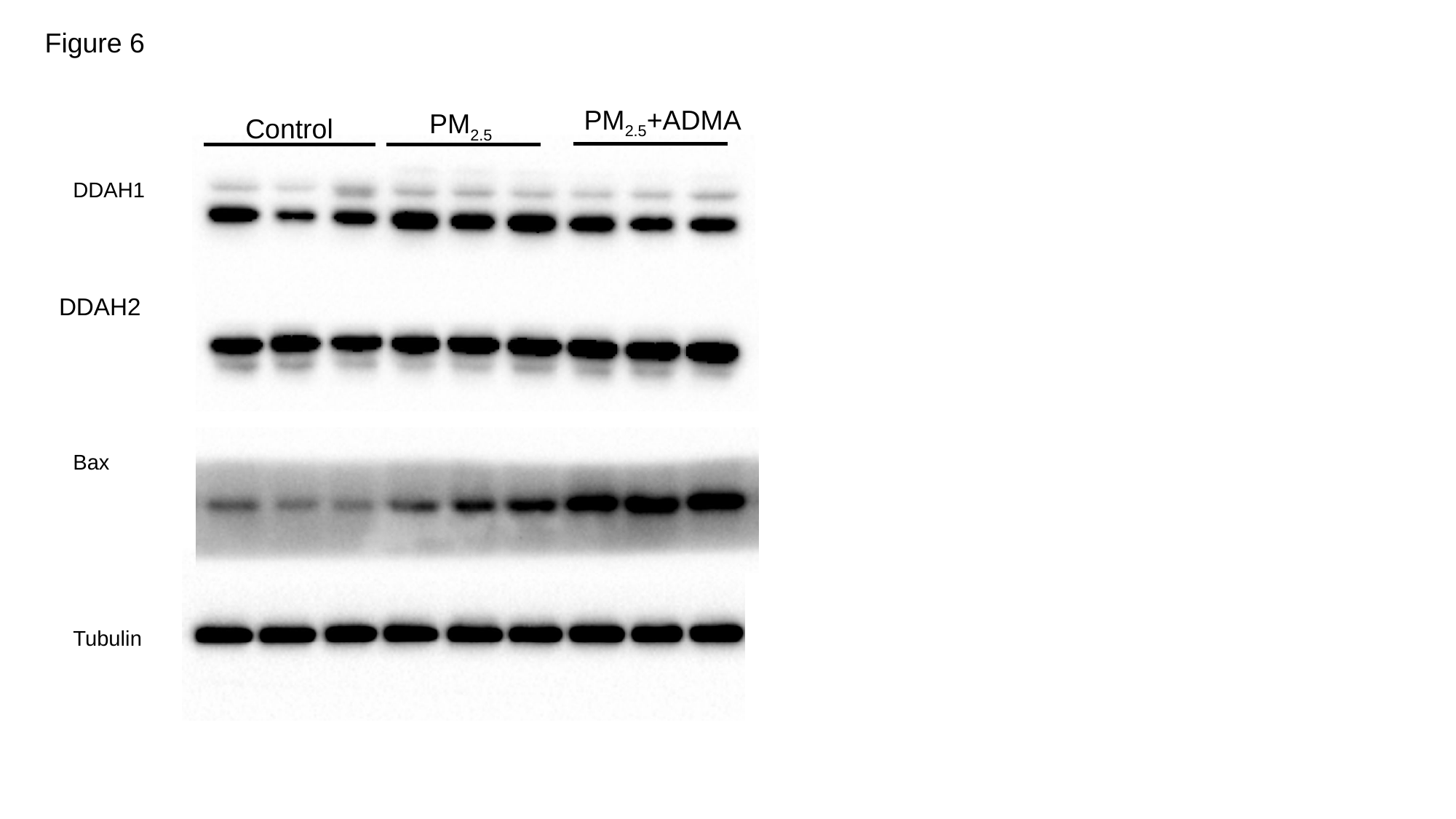

Figure 6
PM2.5+ADMA
PM2.5
Control
DDAH1
DDAH2
Bax
Tubulin

## Slide 5
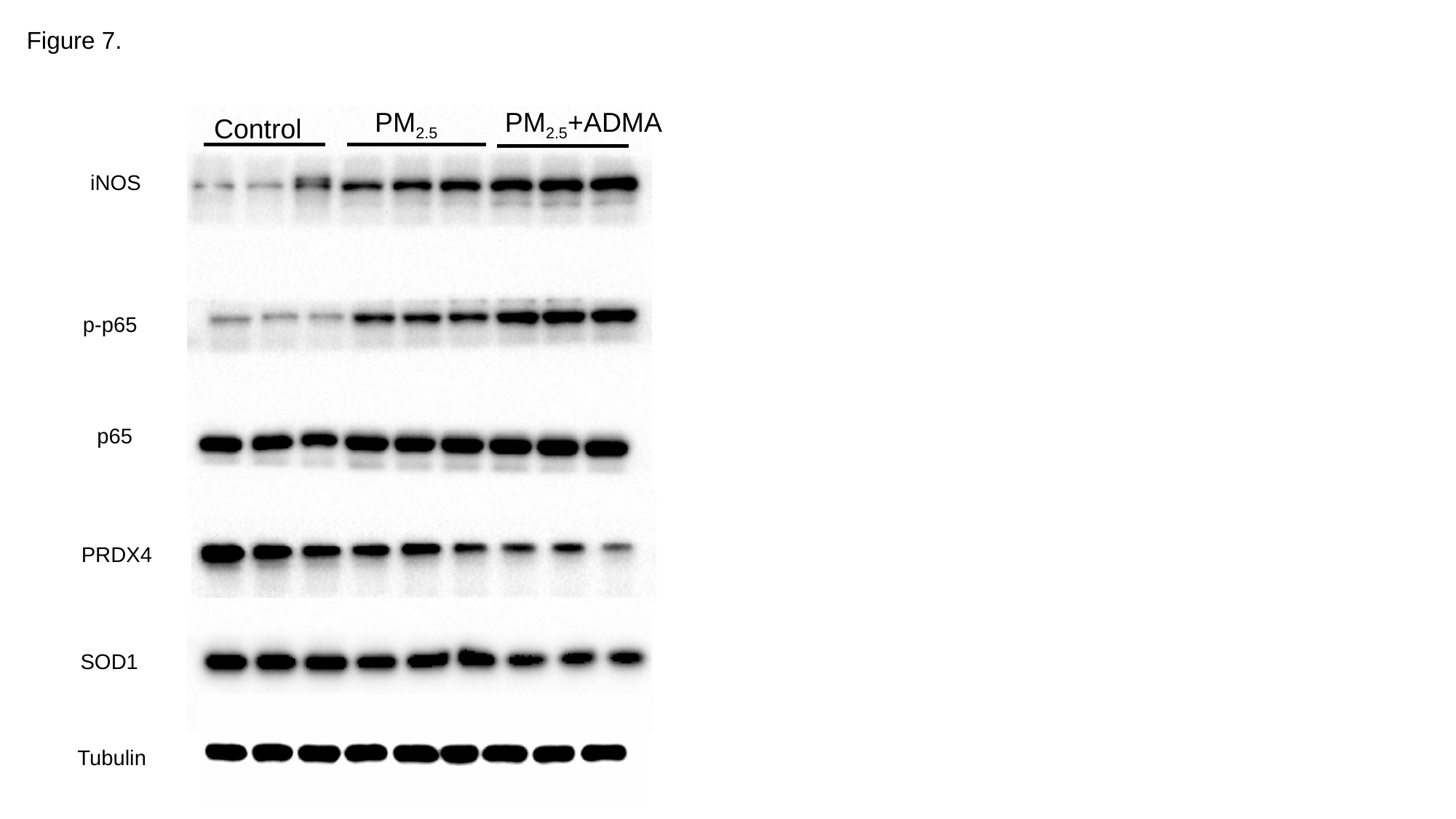

Figure 7.
PM2.5
PM2.5+ADMA
Control
iNOS
p-p65
p65
PRDX4
SOD1
Tubulin

## Slide 6
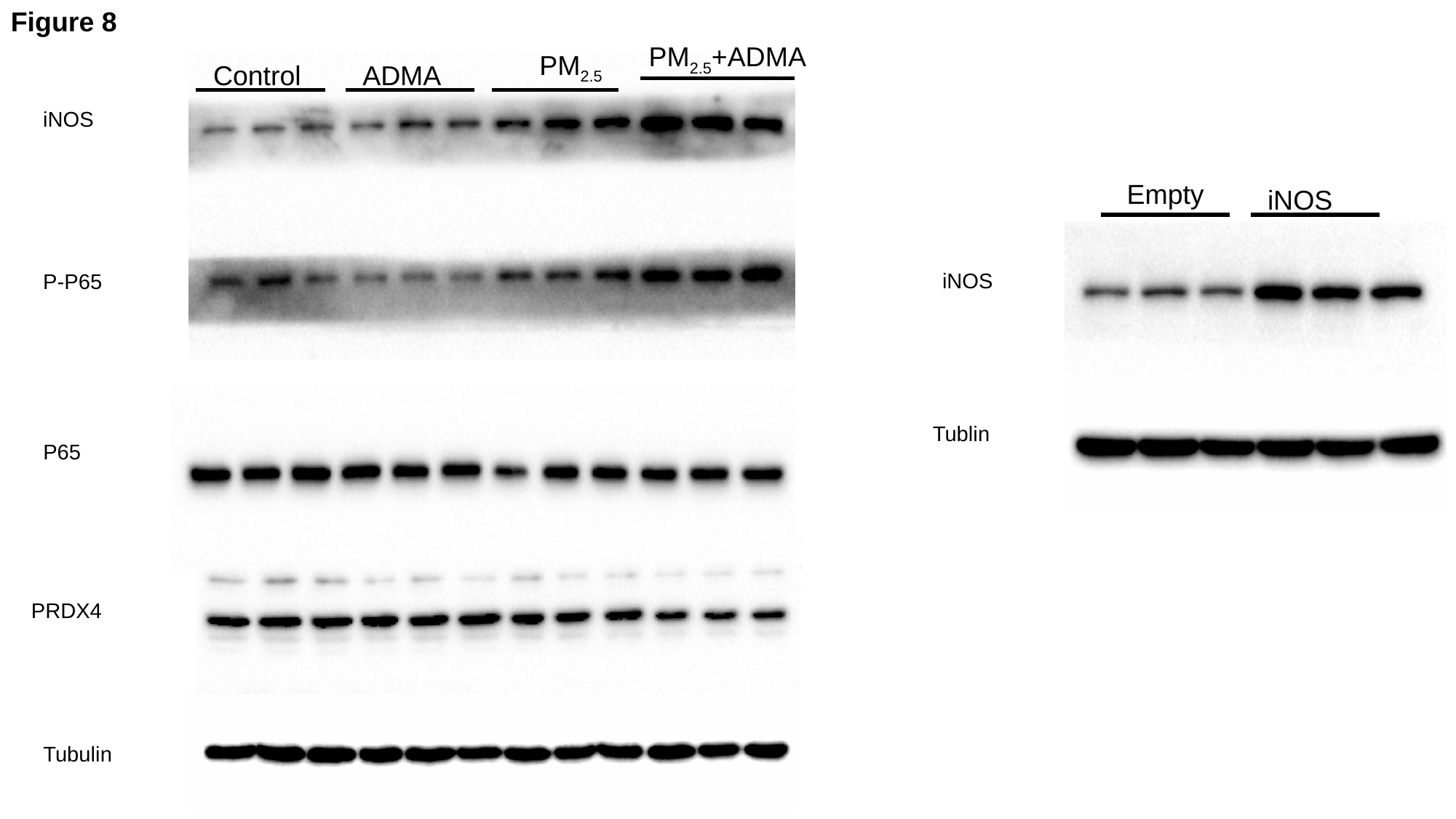

Figure 8
PM2.5+ADMA
PM2.5
Control
ADMA
iNOS
Empty
iNOS
iNOS
P-P65
Tublin
P65
PRDX4
Tubulin
