# Supplementary material for: Dimethylarginine dimethylaminohydrolase 1 protects PM2.5 exposure-induced lung injury in mice by repressing inflammation and oxidative stress
Source: Part Fibre Toxicol. 2022 Oct 14;19:64. doi: 10.1186/s12989-022-00505-7 (PMC9569114; doi:10.1186/s12989-022-00505-7)
Supplement: Supplementary file 2 — Additional file2. Table S1. Metals, soluble inorganic ions, polycyclic aromatic hydrocarbons (PAHs) and carbon in the PM2.5 samples. Table S2. Anatomic data for filter air and long-term PM2.5-exposed mice. Table S3. Anatomic data for control and acute PM2.5-exposed mice. Table S4. Anatomic data for wild type and Ddah1−/− mice after PM2.5 exposure. Table S5. Anatomic data for wild type and DDAH1-TG mice after PM2.5 exposure. Table S6. Detail information for antibodies. Table S7. The quantitative real-time PCR primer information. [file 12989_2022_505_MOESM2_ESM.docx]

**Supplemental File**

**Dimethylarginine dimethylaminohydrolase 1 protects PM_2.5_ exposure-induced lung injury in mice by repressing inflammation and oxidative stress**

Junling Gao^1#^, Tong Lei^1#^, Hongyun Wang^1,2#^, Kai Luo^1^, Yuanli Wang^1^, Bingqing Cui^1^, Zhuoran Yu^1^, Xiaoqi Hu^1^, Fang Zhang^1^, Yingjie Chen^3^, Wenjun Ding^1^*, Zhongbing Lu^1^*

^1^ College of Life Sciences, University of Chinese Academy of Sciences, Beijing, 100049, China.

^2^ Cardiac Regeneration and Ageing Lab, Institute of Cardiovascular Sciences, School of Life Sciences, Shanghai University, Shanghai, 200444, China

^3^ Department of Physiology & Biophysics, University of Mississippi Medical Center, Jackson, MS, 39216, USA

^#^ These authors contributed equally to this work.

* Co-corresponding authors:

Wenjun Ding, PhD

E-mail: [dingwj@ucas.ac.cn](mailto:dingwj@ucas.ac.cn)

Zhongbing Lu, PhD

E-mail: [luzhongbing@ucas.ac.cn](mailto:luzhongbing@ucas.ac.cn)

College of Life Sciences, University of Chinese Academy of Sciences

19A Yuquanlu, Beijing, 100049, China

Fax & Tel: 86-10-69672630

**Table S1. Metals, soluble** **inorganic ions, polycyclic aromatic hydrocarbons (PAHs) and carbon in the PM_2.5_ sample**

| **Metals** | **Concentration(mg/g)** | **Soluble inorganic ions** | **Concentration(mg/g)** |
| --- | --- | --- | --- |
| Ca | 7.643 ± 0.716 | SO_4_^2-^ | 211.6 ± 33.8 |
| Fe | 6.203 ± 0.974 | NO_3_^-^ | 172.1 ± 32.9 |
| Al | 4.931 ± 0.201 | NH_4_^+^ | 61.8 ± 2.3 |
| K | 7.299 ± 0.869 | Cl^-^ | 55.1 ± 10.1 |
| Mg | 1.943 ± 0.570 | Ca^2+^ | 20.3 ± 8.6 |
| Na | 4.253 ± 0.420 | K^+^ | 16 ± 2.9 |
| Zn | 1.137 ± 0.215 | Na^+^ | 6.5 ± 1.5 |
| Pb | 0.887 ± 0.106 | Mg^2+^ | 2.9 ± 0.7 |
| Ti | 0.293 ± 0.141 | NO_2_^-^ | 0.9 ± 0.1 |
| Mn | 0.280 ± 0.049 | F^-^ | 1.7 ± 0.4 |
| Ba | 0.263 ± 0.037 |  |  |
| Cu | 0.175 ± 0.017 | **PAHs** | **Concentration(mg/g)** |
| As | 0.071 ± 0.033 | naphthalene | 0.189 ± 0.066 |
| Sr | 0.073 ± 0.014 | acenaphthylene | 0.066 ± 0.025 |
| Cr | 0.046 ± 0.006 | acenaphthene | 0.032 ± 0.010 |
| Ni | 0.033 ± 0.003 | fluorene | 0.009 ± 0.001 |
| V | 0.016 ± 0.005 | phenanthrene | 0.002 ± 0.001 |
| Cd | 0.013 ± 0.002 | anthracene | 0.068 ± 0.019 |
| Co | 0.003 ± 0.006 | fluoranthene | 0.051 ± 0.019 |
| Mo | 0.011 ± 0.001 | pyrene | 0.062 ± 0.024 |
| Cs | 0.005 ± 0.001 | benzo[a]anthracene | 0.053 ± 0.021 |
|  |  | chrysene | 0.062 ± 0.022 |
| **Carbons** | **Concentration(mg/g)** | benzo[b]fluoranthene | 0.085 ± 0.026 |
| OC | 20.91 ± 2.12 | benzo[k]fluoranthene | 0.052 ± 0.015 |
| EC | 3.44 ± 0.22 | benzo[a]pyrene | 0.075 ± 0.021 |
|  |  | indeno[1,2,3-cd]pyrene | 0.053 ± 0.013 |
|  |  | dibenzo[a,h]anthracene | 0.007 ± 0.002 |
|  |  | coronene | 0.013 ± 0.003 |

Data are mean ± SE. N=4

**Table S2. Anatomic data for filter air and long-term PM_2.5_*-*exposed mice**

| Parameter | FA-6M | PM_2.5_-3M | PM_2.5_-6M |
| --- | --- | --- | --- |
| Number of mice  Body weight(g) | 10  32.46±0.41 | 10  31.98±0.42 | 10 |
|  |  |  | 34.3±0.81 |
| Lung mass(mg) | 158.4±3.05 | 160.3±1.3 | 169.1±5.7 |
| Ratio of lung mass to body weight(mg/g) | 4.90±0.58 | 5.01±0.25 | 4.96±0.95 |

Data are mean ± SE.

**Table S3. Anatomic data for control and acute PM_2.5_*-*exposed mice**

| Parameter | Control | PM_2.5_-2W | PM_2.5_-4W | PM_2.5_-8W |
| --- | --- | --- | --- | --- |
| Number of mice  Body weight(g) | 6  25.83±0.93 | 6  23.2±0.75 | 6 | 6 |
|  |  |  | 25.5±0.53 | 29.5±0.8 |
| Lung mass(mg) | 161.9±7.56 | 207.4±7.84** | 267.4±12.8** | 296.2±6.8** |
| Ratio of lung mass to body weight(mg/g) | 6.27±0.17 | 8.94±0.2** | 10.4±0.39** | 10.6±0.25** |

Data are mean ± SE. ** indicates p<0.01 comparing PM_2.5_ exposure to control conditions

**Table S4. Anatomic data for wild type and *Ddah1*^-/-^ mice after PM_2.5_ exposure**

| Parameter | PM_2.5_-WT | PM_2.5_-*Ddah1*^-/-^ |
| --- | --- | --- |
| Number of mice  Body weight(g) | 8  31.24±0.73 | 8  30.92±0.30 |
| Lung mass(mg) | 162.7±3.5 | 161.7±7.1 |
| Ratio of lung mass to body weight(mg/g) | 5.45±0.16 | 5.45±0.25 |

Data are mean ± SE.

**Table S5. Anatomic data for wild type and DDAH1-TG mice after PM_2.5_ exposure**

| Parameter | PM_2.5_-WT | PM_2.5_-DDAH1-TG |
| --- | --- | --- |
| Number of mice  Body weight(g) | 7  33.66±1.04 | 7  32.14±1.14 |
| Lung mass(mg) | 166.4±6.1 | 167.7±10.8 |
| Ratio of lung mass to body weight(mg/g) | 5.19±0.27 | 5.13±0.41 |

Data are mean ± SE.

**Table S6. Detail information for antibodies**

| **Provider** | **Targeted protein** | **Cat. no.** | **Clone number** | **Source** |
| --- | --- | --- | --- | --- |
| **Cell Signaling Technology (Danvers, MA, USA)** | Bcl-2 | 3498 | D17C4 | rabbit |
|  | Bax | 2772 | N/A | rabbit |
|  | iNOS | 13120 | D6B6S | rabbit |
|  | Phospho-p65 | 3033 | 93H1 | rabbit |
|  | NF-κB p65 | 8242 | D14E12 | rabbit |
| **Abcam PLC (Cambriage, UK)** | DDAH2 | ab184166 | EPR15508(B) | rabbit |
|  | SOD1 | ab51254 | EP1727Y | rabbit |
|  | β-TUBULIN | ab108342 | EPR1330 | rabbit |
| **Signalway Antibody (Greenbelt, MD, USA)** | DDAH1 | 37368 | N/A | rabbit |
|  | PRDX4 | 43303 | N/A | rabbit |
|  | PRMT1 | 49531 | N/A | rabbit |
| **Bioss Biotechnology (Beijing China)** | F4/80 | bs-11182R | N/A | rabbit |
|  | neutrophils | bs-6982R | N/A | rabbit |
| **R&D Systems (Minneapolis, MN, USA)** | ICAM1 | AF796 | N/A | goat |
|  | VACM1 | AF643 | N/A | goat |
| **Merck Millipore (Temecula, CA, USA)** | CD31 | MAB1398Z | 2H8 | hamster |
| **Santa Cruz Biotechnology (Dallas, TX, USA)** | αSMA | sc-32251 | 1A4 | mouse |

**Table S7 The quantitative real-time PCR primer information**

| Genes | Primers | Sequence (5’-3’) |
| --- | --- | --- |
| *18s* | Forward | 5’-TTCTGGCCAACGGTCTAGACAAC-3’ |
|  | Reverse | 5’-CCAGTGGTCTTGGTGTGCTGA-3’ |
| *TNFα* | Forward | 5’- AGGGTCTGGGCCATAGAACT-3’ |
|  | Reverse | 5’- CCACCACGCTCTTCTGTCTAC -3’ |
| *IL-6* | Forward | 5’- ACCAGAGGAAATTTTCAATAGGC-3’ |
|  | Reverse | 5’-TGATGCACTTGCAGAAAACA -3’ |
| *IL-1β* | Forward | 5’- AGGTCAAAGGTTTGGAAGCA -3’ |
|  | Reverse | 5’- TGAAGCAGCTATGGCAACTG-3’ |
| *TGFβ* | Forward | 5’-CAACCCAGGTCCTTCCTAAA -3’ |
|  | Reverse | 5’-GGAGAGCCCTGGATACCAAC-3’ |
| *Collagen I* | Forward | 5’-TAGGCCATTGTGTATGCAGC-3’ |
|  | Reverse | 5'-ACATGTTCAGCTTTGTGGACC-3’ |
| *Collagen III* | Forward | 5’-TAGGACTGACCAAGGTGGCT-3’ |
|  | Reverse | 5’-GGAACCTGGTTTCTTCTCACC-3’ |

**
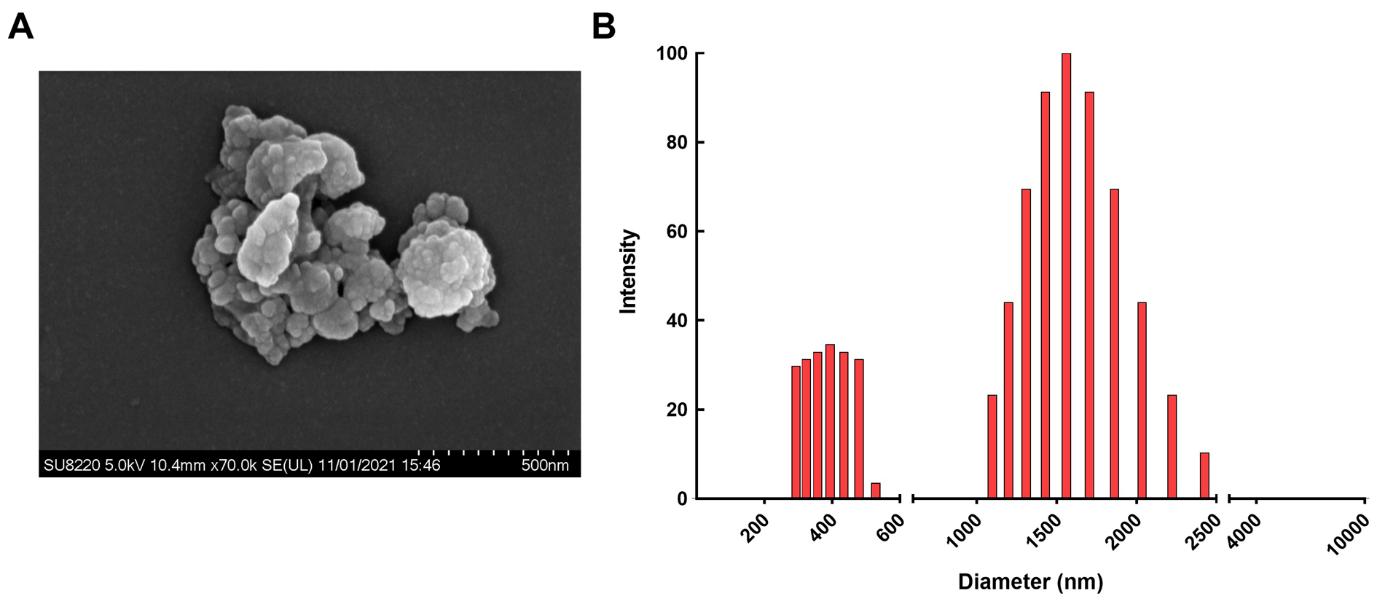
**

**Figure S1. The morphology and size distribution of PM_2.5_.** (A) Scanning electron microscopy image of PM_2.5_. Scale bar = 500 nm. (B) Particle size distribution in the ultrapure water was analyzed by dynamic light scattering.


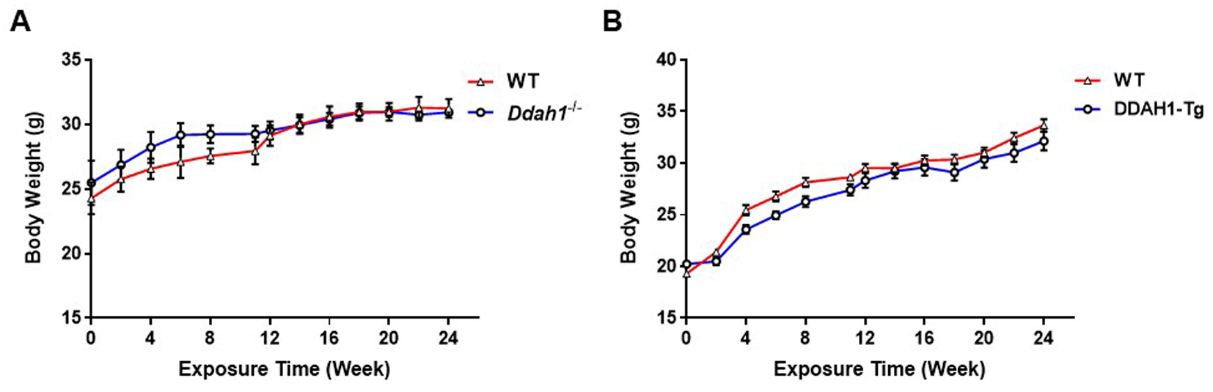


**Figure S2. The body mass curves of PM_2.5_-exposed mice.** During the exposure period, body weight of *Ddah1*^-/-^ mice and wild type (WT) littermates (A), and body weight of human DDAH1 transgenic mice (DDAH1-Tg) and WT littermates were recorded every two weeks. N=8-10, data are presented as mean ± SEM.
